# Regularization by Noise of an Averaged Version of the Navier–Stokes Equations

**DOI:** 10.1007/s10884-023-10255-5

**Published:** 2023-03-28

**Authors:** Theresa Lange

**Affiliations:** Fakultät für Mathematik, Universität Bielefeld, 33501 Bielefeld, Germany

**Keywords:** Transport noise, Regularization by noise, 3D Navier–Stokes equations, 60H15, 60H50, 76D05

## Abstract

In Tao 2016, the author constructs an averaged version of the deterministic three-dimensional Navier–Stokes equations (3D NSE) which experiences blow-up in finite time. In the last decades, various works have studied suitable perturbations of ill-behaved deterministic PDEs in order to prevent or delay such behavior. A promising example is given by a particular choice of stochastic transport noise closely studied in Flandoli et al. 2021. We analyze the model in Tao 2016 in view of these results and discuss the regularization skills of this noise in the context of the averaged 3D NSE.

## Introduction

Consider the Navier–Stokes equations (NSE) on ${\mathbb {R}}^3$ describing the dynamics of an incompressible viscous fluid

1 $$\begin{aligned} \begin{aligned} \partial _t u + (u \cdot \nabla )u + \nabla p&= \varDelta u,\\ \textrm{div} u&= 0,\\ u(0, \cdot )&= u_0, \end{aligned} \end{aligned}$$

with vector-valued velocity field $u:[0,\infty ) \times {\mathbb {R}}^3 \rightarrow {\mathbb {R}}^3$ and scalar-valued pressure field $p:[0,\infty )\times {\mathbb {R}}^3 \rightarrow {\mathbb {R}}$. By a rescaling argument, we may assume unitary viscosity. The global well-posedness of 3D NSE forms a long-standing open problem and has attracted the attention of researchers ever since: it was established in the regime of small initial data (see e.g. [15]) as well as in the hyperdissipative case (replacing $\varDelta$ by $-(-\varDelta )^{\alpha }$, for $\alpha \ge \frac{5}{4}$ see e.g. [13]). The general case remains unresolved; in particular, there exists evidence for blow-up of solutions to variants of (1) as exploited in e.g. [4, 14–17, 19–21]. This note shall focus on the following result presented in [23]: consider the projection of (1) onto the space of divergence-free vector fields, reading

2 $$\begin{aligned} \begin{aligned} \partial _t u&= \varDelta u +B(u,u),\\ u(0,\cdot )&=u_0. \end{aligned} \end{aligned}$$

Here *B* denotes the bilinear Euler operator (see e.g. in [23]) which is symmetric and for sufficiently regular *u* satisfies the cancellation property

3 $$\begin{aligned} \langle B(u,u), u \rangle _{L^2({\mathbb {R}}^3)} = 0 \end{aligned}$$

where $\langle u, v \rangle _{L^2({\mathbb {R}}^3)}:= \int _{{\mathbb {R}}^3} u(x) \cdot v(x) \textrm{d} x$ in $L^2({\mathbb {R}}^3)$. Standard harmonic analysis approaches then use both symmetry and (3) to investigate the behavior of the system’s energy in view of well-posedness. However, [23] demonstrates that such an ansatz will not be successful: the author constructs an explicit, "averaged" version of (2) in which symmetry and cancellation property remain valid, and for which the corresponding system experiences a blow-up in finite time.

In recent years it has been investigated whether for ill-posed deterministic PDEs it is possible to construct a perturbation yielding higher regularity of the corresponding perturbed system. Especially in the case of stochastic perturbations the motive behind such constructions is that noise may have a smoothing effect. This goes under the name of *regularization by noise* and has been analyzed in various settings; in view of the extensive amount of literature available on this account, we merely point out a few overview works such as [6] and [12], or in the context of NSE e.g. [3], and the references therein. A particularly interesting stochastic perturbation is given by a specific type of transport noise which in the context of the vorticity formulation of (1) and similar, more general models proved to yield existence of solutions for arbitrarily long time with large probability, as shown in [7] and [8] respectively. Further work on regularization by transport noise can be found in for instance [1, 5, 9–11, 18].

### Overview of the Results of This Work

The averaged NSE from [23] form an excellent candidate to test the regularization effect of the above noise: though the model explodes in finite time, we want to exploit whether it allows for enough regularity in order for transport noise to delay the blow-up. In [8], this is achieved for systems on the torus if the nonlinearity of the system admits for a continuity, growth and local monotonicity condition which we will recall in Sect. 2. In particular we will formulate the result from [8] in the case of divergence-free vector fields with the proof given in Appendix 1. Consequently, we will consider the averaged NSE on the torus which equally experience the same blow-up statement derived in [23], see Theorem 5 in Sect. 3.1. We proceed by discussing the above delay criteria on the level of regularity classes of the solution. In Sect. 3.2.1, it turns out that (similar to the case of the standard NSE (1)) we do not obtain local existence and uniqueness of solutions to the averaged NSE in $L^2$. In this case the above three conditions cannot be shown to hold true, see Theorem 6. In turn in Sect. 3.2.2, we derive lower bounds on the order of regularity which allow for an analysis as in [8], see Theorem 7 and Theorem 8. Since the blow-up result of [23] was derived in a rather high regularity class, we may therefore still conclude regularization by transport noise in Theorem 9.

### Notation

Let $L^2(\mathbb {K}^d)$ denote the space of square-integrable functions on $\mathbb {K}^d$ with norm $\Vert \cdot \Vert _{L^2(\mathbb {K}^d)}$ where $\mathbb {K}^d = {\mathbb {R}}^d$ or $\mathbb {K}^d = \mathbb {T}^d:= {\mathbb {R}}^d / \mathbb {Z}^d$. Further let $H^{\alpha }(\mathbb {K}^d), \alpha \in {\mathbb {R}},$ denote the Sobolev space endowed with norm $\Vert \cdot \Vert _{H^{\alpha }(\mathbb {K}^d)}:= \left\| (\textrm{Id} -\varDelta )^{\frac{\alpha }{2}}\cdot \right\| _{L^2(\mathbb {K}^d)}$. As already used in the introduction, we will further use $\langle \cdot , \cdot \rangle _{L^2(\mathbb {K}^d)}$ to denote the inner product in $L^2(\mathbb {K}^d)$, and $\langle \cdot , \cdot \rangle$ for the dual pairing of $H^{\alpha }(\mathbb {K}^d)$ and $H^{-\alpha }(\mathbb {K}^d)$. Furthermore let $H^{\alpha }_\textrm{df}(\mathbb {K}^d)$ denote the space of divergence-free functions in $H^{\alpha }(\mathbb {K}^d)$. On the other hand, denoting $L^p(0,T;Z)$ the set of all $u:[0,T]\rightarrow Z$ in $L^p$ for some linear space *Z* with norm $\Vert \cdot \Vert _Z$, we define the fractional Sobolev spaces by

$$\begin{aligned} W^{\beta , p}(0,T;Z):= \left\{ u \in L^p(0,T;Z): \int _0^T \int _0^T \frac{\Vert u(t)-u(s)\Vert _Z^p}{|t-s|^{1+\beta p}}\textrm{d}t \textrm{d}s < \infty \right\} \end{aligned}$$

for $\beta \in (0,1), p >1$. If it exists, we will denote by $\mathcal {F}_{\mathbb {K}^d}f$ the Fourier transform of a function *f* on $\mathbb {K}^d$. Whenever it is clear from the context, we drop the $\mathbb {K}^d$ in the notation. Finally, let $\textrm{supp}$ denote the support of a function, and let $x \lesssim y$ denote $x \le C y$ for some constant $C>0$.

## Main Ingredients

This section shall serve the purpose of bringing together the various objects considered in this work. It will mainly consist of repetitions of external work and will set the notation throughout.

### The Averaged NSE

The main idea in [23] is to construct a modification of the projected NSE (2) which on the one hand preserves energy as well as symmetry and cancellation property of the nonlinearity, and on the other hand experiences a blow-up. In order to do so, consider a suitable frequency decomposition of the projected NSE and "average out", i.e. eliminate a carefully selected choice of frequencies, resulting in a system of the following form

4 $$\begin{aligned} \begin{aligned} \partial _t u&= \varDelta u +C(u,u),\\ u(0,\cdot )&= u_0, \end{aligned} \end{aligned}$$

where *C* is called a *local cascade operator*, introduced below. Thus allowing only for localized frequency interactions gives rise to a concrete blow-up mechanism. Considering the subcritical case of mild solutions in the regularity class $H_\textrm{df}^{10}({\mathbb {R}}^3)$, the main result in [23] is as follows:

#### Theorem 1

(cf. [23, Theorem 3.3]) There exist a symmetric local cascade operator *C* satisfying the cancellation property, and a divergence-free vector field $u_0$ such that there does not exist any global mild solution $u:[0,\infty )\rightarrow H_\textrm{df}^{10}({\mathbb {R}}^3)$ to (4).

The construction of such an operator *C* in [23] is inspired by the work of [14] in case of the dyadic hyperdissipative NSE: heuristically, a solution *u* to the projected NSE (2) can be approximated by a wavelet decomposition of the form

5 $$\begin{aligned} \sum _n u_n (t)\psi _n(x) \end{aligned}$$

for a suitable orthonormal basis $\{\psi _n\}$ in $L^2({\mathbb {R}}^3)$, and the wavelet coefficients $u_n$ evolve according to the following ODEs

6 $$\begin{aligned} \partial _t u_n = 2^{\frac{5n}{2}} u_{n-1}^2 -2^{2 n}u_n - 2^{\frac{5(n+1)}{2}} u_n u_{n+1}. \end{aligned}$$

Hence the corresponding energy equation reads

$$\begin{aligned} \partial _t \left( \frac{1}{2} u_n^2\right) = 2^{\frac{5n}{2}} u_{n-1}^2u_n -2^{2n}X_n^2 - 2^{\frac{5(n+1)}{2}} u_n^2 u_{n+1}, \end{aligned}$$

encoding a ’low-to-high-frequency-cascade’: the energy from the previous scale $n-1$ enters scale *n* and, apart from some dissipated portion, will be completely transported to the next scale $n+1$. This system experiences a blow-up in $H^{2+\epsilon }({\mathbb {R}}^3)$ for small $\epsilon >0$ and dissipation exponent $\alpha <\frac{1}{4}$; however, in [2] it has been shown that in the dissipation range containing the standard NSE, the corresponding model dissipates energy fast enough to prevent such a blow-up. The construction in [23] allows for a decomposition (5) in such a way that the system of coefficients $u_n$ captures an additional time delay in which energy first accumulates at one scale and is then abruptly transported to the next. This way the energy cascade outruns the dissipation and yields a blow-up in finite time. Let us now give the precise formulation of an operator *C* enabling such behaviour:

#### Definition 1

(cf. [23, Section 4]) Let $\epsilon _0 \in (0,1)$ and $m\in {\mathbb {N}}$. Furthermore let $B_1,\ldots ,B_m$ be balls in the annulus $\{\xi \in {\mathbb {R}}^3: 1< |\xi |<1+\frac{\epsilon _0}{2}\}$ such that $B_1,\ldots ,B_m,-B_1,\ldots ,-B_m$ are disjoint. For $n\in \mathbb {Z}$ and $i\in \{1,\ldots ,m\}$, let $\psi _{i,n}:{\mathbb {R}}^3\rightarrow {\mathbb {R}}^3$ be rescaled $L^2$-functions with

$$\begin{aligned}\psi _{i,n}(\cdot ):= (1+\epsilon _0)^{\frac{3n}{2}}\psi _i((1+\epsilon _0)^n \cdot )\end{aligned}$$

where $\psi _i \in H_\textrm{df}^{10}({\mathbb {R}}^3)$ are Schwartz functions with Fourier transform supported on $B_i \cup -B_i$ and normalized to $\Vert \psi _i\Vert _{L^2}=1$. Let $S=\{(0,0,0),(1,0,0),(0,1,0),(0,0,1)\}$ and $\alpha _{i_1,i_2,i_3,\mu _1,\mu _2,\mu _3}\in {\mathbb {R}}$ be bounded structure constants where $i_1,i_2,i_3 \in \{1,\ldots ,m\}$ and $(\mu _1,\mu _2,\mu _3)\in S$. Then a *local cascade operator* $C:H_\textrm{df}^{10}({\mathbb {R}}^3) \times H_\textrm{df}^{10}({\mathbb {R}}^3) \rightarrow H_\textrm{df}^{-10}({\mathbb {R}}^3)$ is defined by

7 $$\begin{aligned} \begin{aligned} C(u,v):=\sum _{n \in \mathbb {Z}}\sum _{(i_1,i_2,i_3,\mu _1,\mu _2,\mu _3)\in \{1,\ldots ,m\}^3\times S}&\alpha _{i_1,i_2,i_3,\mu _1,\mu _2,\mu _3} (1+\epsilon _0)^{\frac{5n}{2}}\\ {}&\langle u, \psi _{i_1,n+\mu _1}\rangle _{L^2} \langle v, \psi _{i_2,n+\mu _2} \rangle _{L^2} \psi _{i_3,n+\mu _3} \end{aligned} \end{aligned}$$

for $$u,v \in H_\textrm{df}^{10}({\mathbb {R}}^3)$$.

#### Remark 1

In the following we will rather use the short hand notation $\sum _{n,i,\mu }$ as well as $\alpha _{i,\mu }$.

As done in [23], requiring the symmetry condition

$$\begin{aligned} \alpha _{i_1,i_2,i_3,\mu _1,\mu _2,\mu _3} = \alpha _{i_2,i_1,i_3,\mu _2,\mu _1,\mu _3} \end{aligned}$$

as well as the cancellation condition

$$\begin{aligned} \sum _{\{a,b,c\}=\{1,2,3\}} \alpha _{i_a,i_b,i_c,\mu _a,\mu _b,\mu _c} =0 \end{aligned}$$

for all $i_1,i_2,i_3 \in \{1,\ldots ,m\}$ and $(\mu _1,\mu _2,\mu _3)\in S$, this ensures that *C* is symmetric and satisfies the cancellation property

$$\begin{aligned} \langle C(u,u),u\rangle _{L^2}=0\quad \forall u \in H_\textrm{df}^{10}({\mathbb {R}}^3). \end{aligned}$$

Next consider the corresponding Cauchy problem

8 $$\begin{aligned} \begin{aligned} \partial _t u&= \varDelta u +C(u,u),\\ u(0,\cdot )&= u_0:= \psi _{1,n_0}, \end{aligned} \end{aligned}$$

for some $n_0 \in {\mathbb {N}}$ sufficiently large, and assume that there exists a mild solution $u:[0,\infty ) \rightarrow H_\textrm{df}^{10}({\mathbb {R}}^3)$. Then the following holds:

#### Lemma 1

(cf. [23, Lemma 4.1]) For each $n\in \mathbb {Z}, t \ge 0$ and $i\in \{1,\ldots ,m\}$ define

$$\begin{aligned} \left( \mathcal {F}_{{\mathbb {R}}^3}u_{i,n}(t)\right) (\xi )&:= \left( \mathcal {F}_{{\mathbb {R}}^3}u(t)\right) (\xi )\mathbbm {1}_{\{ \xi \in (1+\epsilon _0)^n(B_i \cup -B_i)\}},\\ X_{i,n}(t)&:= \langle u(t), \psi _{i,n} \rangle _{L^2} = \langle u_{i,n}(t),\psi _{i,n}\rangle _{L^2},\\ E_{i,n}&:= \frac{1}{2} \Vert u_{i,n}(t)\Vert _{L^2}^2, \end{aligned}$$

then

1. it holds
  9 $$\begin{aligned} \sup _{t\in [0,T]}\sup _{n\in \mathbb {Z}}\sup _{i\in \{1,\ldots ,m\}} \left( 1+(1+\epsilon _0)^{10n}\right) |X_{i,n}(t)|<\infty \end{aligned}$$
  and
  10 $$\begin{aligned} \sup _{t\in [0,T]}\sup _{n\in \mathbb {Z}}\sup _{i\in \{1,\ldots ,m\}} \left( 1+(1+\epsilon _0)^{10n}\right) |E_{i,n}(t)|<\infty \end{aligned}$$
  for all $$T \in (0,\infty )$$,
2. for any $$n \in \mathbb {Z}, i\in \{1,\ldots ,m\}$$
  11 $$\begin{aligned} E_{i,n}(0)&= \frac{1}{2}X_{i,n}(0)^2,\end{aligned}$$
  12 $$\begin{aligned} X_{i,n}(0)&= \mathbbm {1}_{\{(i,n)=(1,n_0)\}}, \end{aligned}$$
3. for any $$n \in \mathbb {Z}, i\in \{1,\ldots ,m\}$$
  13 $$\begin{aligned} \begin{aligned} \partial _t X_{i,n}&= \sum _{i_1,i_2\in \{1,\ldots ,m\}}\sum _{\mu \in S} \alpha _{i_1,i_2,i,\mu } (1+\epsilon _0)^{\frac{5(n-\mu _3)}{2}} X_{i_1,n-\mu _3+\mu _1}X_{i_2,n-\mu _3+\mu _2}\\ {}&\quad + O\left( (1+\epsilon _0)^{2n}\sqrt{E_{i,n}}\right) \end{aligned} \end{aligned}$$
  and
  14 $$\begin{aligned} \partial _t E_{i,n} \le \sum _{i_1,i_2\in \{1,\ldots ,m\}}\sum _{\mu \in S} \alpha _{i_1,i_2,i,\mu } (1+\epsilon _0)^{\frac{5(n-\mu _3)}{2}} X_{i_1,n-\mu _3+\mu _1}X_{i_2,n-\mu _3+\mu _2}X_{i,n}, \end{aligned}$$
4. for any $$n \in \mathbb {Z}, i\in \{1,\ldots ,m\}$$
  15 $$\begin{aligned} \frac{1}{2}X_{i,n}(t)^2 \le E_{i,n}(t) \le \frac{1}{2}X_{i,n}(t)^2 + O\left( (1+\epsilon _0)^{2n}\int _0^t E_{i,n}(s)\textrm{d}s\right) , \end{aligned}$$
5. it holds
  16 $$\begin{aligned} X_{i,n}(t)=0=E_{i,n}(t) \end{aligned}$$
  for all $$n<n_0, i\in \{1,\ldots ,m\}, t\ge 0$$.

Observe that up to the *O*-terms, (13) is of the form (6) and existence of a global mild solution *u* implies boundedness of the $X_{i,n}$ as formalized in (9). Thus in order to prove Theorem 1, the author constructs a sequence $(X_{i,n})$ violating Lemma 1 which specifies a blow-up in $H_\textrm{df}^{10}$ (cf. [23, Theorem 4.2] as well as the construction in [23, Section 6]).

### Regularization by Transport Noise

In this section, we shall introduce the specific choice of transport noise (cf. [7, Section 2] and [8, Section 1.2]): on the *d*-dimensional torus $\mathbb {T}^d$ consider the following noise

17 $$\begin{aligned} \frac{\sqrt{C_d \nu }}{\Vert \theta \Vert _{\ell ^2}}\sum _{k \in \mathbb {Z}_0^d}\sum _{i=1}^{d-1}\theta _k \Pi ((\sigma _{k,i}\cdot \nabla )\cdot )W^{k,i}. \end{aligned}$$

Here $C_d =\frac{d}{d-1}, d \ge 2, \nu >0$, $\Pi$ is the Leray projection and the individual components are as follows: let $\ell ^2 = \ell ^2(\mathbb {Z}_0^d)$ denote the space of square-summable sequences indexed by $\mathbb {Z}_0^d=\mathbb {Z}^d{\setminus }\{0\}$ and choose a sequence $\theta =(\theta _k)_{k \in \mathbb {Z}_0^d} \in \ell ^2$ with finitely many non-zero components such that $\theta$ satisfies a symmetry condition

$$\begin{aligned} \theta _k = \theta _l \quad \forall k, l \in \mathbb {Z}_0^d,\quad |k|=|l|. \end{aligned}$$

Further, let $\{ \sigma _{k,i}: k \in \mathbb {Z}_0^d, i=1,\ldots ,d-1\}$ be periodic divergence-free smooth vector fields forming a complex orthonormal system of the space

18 $$\begin{aligned} H_{\mathbb {C}}=\left\{ v \in L^2(\mathbb {T}^d,\mathbb {C}^d):\quad \int _{\mathbb {T}^d} v \textrm{d} x = 0, \textrm{div} v =0\right\} \end{aligned}$$

and which are defined as follows:

$$\begin{aligned} \sigma _{k,i}(x) = a_{k,i}e^{2\pi \textrm{i}k\cdot x}, \quad x \in \mathbb {T}^d, k\in \mathbb {Z}_0^d, i=1,\ldots ,d-1. \end{aligned}$$

Here $\textrm{i}$ denotes the imaginary unit and considering a partition $\mathbb {Z}_{+}^d, \mathbb {Z}_{-}^d$ of $\mathbb {Z}_0^d$ such that $\mathbb {Z}_0^d = \mathbb {Z}_{+}^d \cup \mathbb {Z}_{-}^d, \mathbb {Z}_{+}^d = - \mathbb {Z}_{-}^d$, choose for any $k \in \mathbb {Z}_{+}^d$ the set $\{a_{k,i}:i=1,\ldots ,d-1\}$ to be an ONB of $k^{\perp }:= \{y \in {\mathbb {R}}^d: y \cdot k =0\}$, and define $a_{k,i} = a_{-k,i}$ for any $k \in \mathbb {Z}_{-}^d$.

Finally let $\{W^{k,i}:k \in \mathbb {Z}_0^d,i=1,\ldots ,d-1\}$ be a family of complex Brownian motions on a probability space $(\Omega , \mathcal {F},\mathbb {P})$ such that

19 $$\begin{aligned} \overline{W^{k,i}} = W^{-k,i} \end{aligned}$$

and their cross-variation satisfies

20 $$\begin{aligned} \left[ W^{k,i},W^{l,j}\right] _t = 2t \delta _{k+l}\delta _{i-j} \quad \forall k,l \in \mathbb {Z}_0^d, i,j \in \{1,\ldots ,d-1\} \end{aligned}$$

in order for $W^{k,i}$ and $W^{l,j}$ to be independent whenever $k \ne \pm l$ and $i \ne j$.

**Example**: In [7], the authors consider a family $\{B^{k,i}:k \in \mathbb {Z}_0^d,i=1,\ldots ,d-1\}$ of standard real-valued Brownian motions and define for $k \in \mathbb {Z}_{+}^d$

$$\begin{aligned} W^{k,i}:= B^{k,i} +\textrm{i}B^{-k,i} \end{aligned}$$

and for $$k \in \mathbb {Z}_{-}^d$$

$$\begin{aligned} W^{k,i}:= B^{k,i} -\textrm{i}B^{-k,i}. \end{aligned}$$

It is easy to check that $\{W^{k,i}:k \in \mathbb {Z}_0^d, i=1,\ldots ,d-1\}$ then satisfy (19) and (20).

#### The Vorticity Formulation of NSE

For $d=3$, the vorticity $\xi := \nabla \times u$ of the standard NSE (1) evolves according to

21 $$\begin{aligned} \partial _t \xi +\mathcal {L}_u \xi =\varDelta \xi \end{aligned}$$

with Lie derivative $\mathcal {L}_u \xi = (u\cdot \nabla )\xi - (\xi \cdot \nabla )u$ consisting of a transport and a vortex stretching term, respectively. As discussed in [7], we may heuristically recover the form of noise (17) here when separating the vorticity into large-scale and small-scale component and treating the later as a random perturbation of the former. The small-scale vortex stretching term, however, complicates the regularization-by-noise analysis but it is shown in [7] that the transport term on its own already has sufficient regularization skills. More precisely, let $B_H(R_0)$ denote the ball of radius $R_0$ in the real subspace *H* of $H_{\mathbb {C}}$, then the authors of [7] are able to show the following result:

##### Theorem 2

(cf. [7, Corollary 1.5]) For $R_0 >0$, $T>0$, and $\epsilon >0$, there exists $\theta \in \ell ^2$ such that for all $\xi _0 \in B_H(R_0)$

22 $$\begin{aligned} \text {d} \xi +\mathcal {L}_u \xi \text {d}t = \varDelta \xi \text {d}t + \frac{\sqrt{C_3 \nu }}{\Vert \theta \Vert _{\ell ^2}}\sum _{k \in \mathbb {Z}_0^3}\sum _{i=1}^{2}\theta _k \Pi ((\sigma _{k,i}\cdot \nabla )\xi )\circ \text {d}W^{k,i} \end{aligned}$$

admits a unique strong solution up to time *T* with probability no less than $1-\epsilon$.

For the proof rewrite the Stratonovich equation (22) into its corresponding Itô-formulation which by the above choice of parameters is of the form

23 $$\begin{aligned} \text {d} \xi +\mathcal {L}_u \xi \text {d}t = \left( \varDelta \xi + S_{\theta }(\xi )\right) \text {d}t + \frac{\sqrt{C_3 \nu }}{\Vert \theta \Vert _{\ell ^2}}\sum _{k \in \mathbb {Z}_0^3}\sum _{i=1}^{2}\theta _k \Pi ((\sigma _{k,i}\cdot \nabla )\xi ) \text {d}W^{k,i} \end{aligned}$$

with Itô-Stratonovich correction denoted by $S_{\theta }(\xi )$. Then they show that there exists a suitable choice of sequence $(\theta ^N)_{N \in {\mathbb {N}}}$ such that in a suitable sense specified in [7], in the limit of $N\rightarrow \infty$ the martingale part in (23) vanishes and

$$\begin{aligned} \lim _{N \rightarrow \infty } S_{\theta ^N}(\xi ) = \frac{3}{5}\nu \varDelta \xi . \end{aligned}$$

Hence obtain the limiting equation

24 $$\begin{aligned} \partial _t \xi + \mathcal {L}_u \xi = \left( 1+\frac{3}{5}\nu \right) \varDelta \xi \end{aligned}$$

and the claim then follows by using existence of a unique global strong solution to (24) for large enough $\nu$.

#### Criteria for Delayed Blow-up

In the case of $\mathbb {T}^d$, $d \ge 2$, consider systems of more general form, namely

25 $$\begin{aligned} \begin{aligned} \partial _t u&= -(-\varDelta )^{\alpha } u + F(u),\\ u(0,\cdot )&= u_0, \end{aligned} \end{aligned}$$

for $\alpha \ge 1$ and a fixed initial condition $u_0 \in L^2(\mathbb {T}^d)$. Regularization by transport noise is obtained under the following structural assumptions:

(H1): *Continuity*: There exist $\beta _1\ge 0$ and $\eta \in (0,\alpha )$ such that $F:H^{\alpha - \eta } (\mathbb {T}^d)\rightarrow H^{-\alpha }(\mathbb {T}^d)$ is continuous and
  $$\begin{aligned} \Vert F(u)\Vert _{H^{-\alpha }(\mathbb {T}^d)} \lesssim \left( 1+\Vert u\Vert _{L^2(\mathbb {T}^d)}^{\beta _1}\right) \left( 1+\Vert u\Vert _{H^{\alpha }(\mathbb {T}^d)}\right) . \end{aligned}$$
(H2): *Growth*: There exist $\beta _2 \ge 0$ and $\gamma _2 \in (0,2)$ such that
  $$\begin{aligned} |\langle F(u),u\rangle | \lesssim \left( 1+\Vert u\Vert _{L^2(\mathbb {T}^d)}^{\beta _2}\right) \left( 1+\Vert u\Vert _{H^{\alpha }(\mathbb {T}^d)}^{\gamma _2}\right) . \end{aligned}$$
(H3): *Local monotonicity*: There exist $\beta _3, \kappa \ge 0$, $\gamma _3 \in (0,2)$ such that $\beta _3 + \gamma _3 \ge 2$, $\kappa + \gamma _3 \le 2$ and
  $$\begin{aligned} \begin{aligned}&|\langle u-v, F(u) - F(v) \rangle |\lesssim \Vert u-v\Vert _{L^2(\mathbb {T}^d)}^{\beta _3} \Vert u-v\Vert _{H^{\alpha }(\mathbb {T}^d)}^{\gamma _3}\left( 1+\Vert u\Vert _{H^{\alpha }(\mathbb {T}^d)}^{\kappa } + \Vert v\Vert _{H^{\alpha }(\mathbb {T}^d)}^{\kappa }\right) . \end{aligned} \end{aligned}$$
(H4): *Admissible initial conditions*: There exists $\mathcal {K} \subset L^2(\mathbb {T}^d)$ convex, closed and bounded with the following property: for any $T>0$, we can find $\nu >0$ big enough such that the deterministic Cauchy problem
  26 $$\begin{aligned} \begin{aligned} \partial _t u&= -(-\varDelta )^{\alpha }u + \nu \varDelta u +F(u),\\ u(0,\cdot )&=u_0, \end{aligned} \end{aligned}$$
  admits a global solution $u:=u(\cdot ; u_0, \nu ) \in L^2(0,T; H^{\alpha }(\mathbb {T}^d))\cap C([0,T]; L^2(\mathbb {T}^d))$ for any $u_0 \in \mathcal {K}$, and moreover
  27 $$\begin{aligned} \sup _{u_0 \in \mathcal {K}} \sup _{t \in [0,T]} \Vert u(t; u_0, \nu )\Vert _{L^2(\mathbb {T}^d)} < \infty . \end{aligned}$$

Given a deterministic $u_0 \in L^2(\mathbb {T}^d)$, let $\tau (u_0, \nu , \theta )$ denote the random maximal time of existence of solutions $u(t; u_0, \nu , \theta )$ to

28 $$\begin{aligned} \begin{aligned} \textrm{d} u&= (-(-\varDelta )^{\alpha } u + F(u))\textrm{d} t + \frac{\sqrt{C_d \nu }}{\Vert \theta \Vert _{\ell ^2}}\sum _{k \in \mathbb {Z}_0^d}\sum _{i=1}^{d-1}\theta _k (\sigma _{k,i}\cdot \nabla )u \circ \textrm{d}W^{k,i},\\ u(0,\cdot )&= u_0, \end{aligned} \end{aligned}$$

with trajectories in $C([0,T];L^2(\mathbb {T}^d))$. Then

##### Theorem 3

(cf. [8, Theorem 1.4]) Assume *F* satisfies (H1)–(H3) and $\mathcal {K} \subset L^2(\mathbb {T}^d)$ satisfies (H4). Then for arbitrary large time $T\in (0,\infty )$, $\nu = \nu (T) >0$ as in (H4) and arbitrary small $\epsilon >0$, there exists $\theta \in \ell ^2$ such that

29 $$\begin{aligned} \mathbb {P}\left[ \tau (u_0, \nu , \theta )\ge T\right] > 1- \epsilon \quad \forall u_0 \in \mathcal {K}. \end{aligned}$$

##### Remark 2

1. Assuming exponential decay of the $L^2(\mathbb {T}^3)$-norm of the solution to the deterministic system (26) as well as existence of a pathwise unique global solution to (28) for small initial conditions, then Theorem 3 may even be extended to hold for infinite time horizon (cf. [8, Theorem 1.4]).
2. By [8, Remark 1.3, (iii)], if *F* preserves the space of mean-zero functions, then considering the dynamics restricted to this closed subspace of $L^2(\mathbb {T}^d)$ as well as for any fixed constant $R\ge 0$, a suitable choice for $\mathcal {K}$ is
  30 $$\begin{aligned} \mathcal {K} = \left\{ f \in L^2(\mathbb {T}^d): \int _{\mathbb {T}^d} f \textrm{d} x = 0, \Vert f\Vert _{L^2} \le R \right\} . \end{aligned}$$
3. A useful implication of (H2) is given in [8, Remark 3.5]: for sufficiently small parameter $\delta >0$, standard interpolation yields (H2’) There exist $\tilde{\beta }_2>0$ and $\tilde{\gamma }_2 <2$ such that
  31 $$\begin{aligned} |\langle F(u), u\rangle | \lesssim \left( 1+\Vert u\Vert _{H^{\alpha }(\mathbb {T}^d)}^{\tilde{\gamma }_2}\right) \left( 1+\Vert u\Vert _{H^{-\delta }(\mathbb {T}^d)}^{\tilde{\beta }_2}\right) . \end{aligned}$$
4. By [8, Remark 1.3, (ii)], hypothesis (H3) can be further generalized to (H3’) There exist $N \in {\mathbb {N}}$ and non-negative parameters $\beta _3^j, \gamma _3^j, \kappa _j, \kappa _j'$, $j=1,\ldots ,N$ such that $\gamma _3^j \in (0,2), \beta _3^j + \gamma _3^j \ge 2, \gamma _3^j + \kappa _j \le 2$ for all *j* and
  32 $$\begin{aligned} \begin{aligned}&|\langle u-v, F(u) - F(v) \rangle |\\&\lesssim \sum _{j=1}^N \Vert u-v\Vert _{L^2(\mathbb {T}^d)}^{\beta _3^j}\Vert u-v\Vert _{H^{\alpha }(\mathbb {T}^d)}^{\gamma _3^j}\\&\qquad \left( 1+\Vert u\Vert _{L^2(\mathbb {T}^d)}^{\kappa _j'}+\Vert v\Vert _{L^2(\mathbb {T}^d)}^{\kappa _j'}\right) \left( 1+\Vert u\Vert _{H^{\alpha }(\mathbb {T}^d)}^{\kappa _j}+\Vert v\Vert _{H^{\alpha }(\mathbb {T}^d)}^{\kappa _j}\right) . \end{aligned} \end{aligned}$$

Observe that solutions to (25) need not be divergence-free, hence the noise in (28) does not contain the Leray projection $\Pi$ (compare with (17)). In the course of this note, we will, however, be in the setting of divergence-free systems. Thus let

$$\begin{aligned} \mathcal {D}:= \left\{ u \in L^2(\mathbb {T}^d):\quad \textrm{div} u =0\right\} \end{aligned}$$

and $\tilde{\tau }(u_0, \nu , \theta )$ denote the analogon to $\tau (u_0, \nu , \theta )$ for

33 $$\begin{aligned} \begin{aligned} \textrm{d} u&= (-(-\varDelta )^{\alpha } u + F(u))\textrm{d} t + \frac{\sqrt{C_d \nu }}{\Vert \theta \Vert _{\ell ^2}}\sum _{k \in \mathbb {Z}_0^d}\sum _{i=1}^{d-1}\theta _k \Pi ((\sigma _{k,i}\cdot \nabla )u) \circ \textrm{d}W^{k,i},\\ u(0,\cdot )&= u_0. \end{aligned} \end{aligned}$$

Then using the tools of [7] in the proof of Theorem 3 gives the following adapted result:

##### Theorem 4

Additionally to the assumptions in Theorem 3, let *F* preserve $\mathcal {D}$. Then for arbitrary large time $T>0$ and arbitrary small $\epsilon >0$, there exists $\theta \in \ell ^2$ such that

34 $$\begin{aligned} \mathbb {P}\left[ \tilde{\tau }(u_0, \nu , \theta )\ge T\right] > 1- \epsilon \quad \forall u_0 \in \mathcal {K}\cap \mathcal {D}. \end{aligned}$$

The proof shall be given in Appendix 1.

## Main Results

In this section, we shall bring together the components introduced in Sect. 2. Since the analysis for the transport noise in Sect. 2.2.2 currently works only on the torus, we shall first check whether the analysis from [23] can be transferred to $\mathbb {T}^3$.

### The Averaged NSE on $\mathbb {T}^3$

Consider the following periodization of the functions $\psi _{i,n}$ in Definition 1:

35 $$\begin{aligned} \psi _{i,n}^\textrm{per}(x):=\sum _{l \in \mathbb {Z}^3} \psi _{i,n}(x+l). \end{aligned}$$

We observe the following: since $\psi _i$ is a Schwartz function on ${\mathbb {R}}^3$, we obtain for all $N \in {\mathbb {N}}$ that

36 $$\begin{aligned} \sup _{x\in \mathbb {T}^3} | \psi _{i, n} (x + l) |^2 (1 + | x + l |)^N \lesssim _N 1 \quad \forall l \in \mathbb {Z}^3, \end{aligned}$$

hence let $N > 3$, then

$$\begin{aligned} \sum _{l \in \mathbb {Z}^3} | \psi _{i, n} (x + l) |^2 \lesssim _N \sum _{l \in \mathbb {Z}^3} (1 + | x + l |)^{- N} < \infty \end{aligned}$$

and

$$\begin{aligned} \int _{\mathbb {T}^3} (1 + | x + l |)^{- N} \textrm{d} x \end{aligned}$$

is summable. Therefore (35) is well-defined and we may exchange integration and summation to obtain for $k \in \mathbb {Z}^3$

37 $$\begin{aligned} \begin{aligned} (\mathcal {F}_{\mathbb {T}^3} \psi ^\textrm{per}_{i, n}) (k)&= \int _{\mathbb {T}^3} \sum _{l \in \mathbb {Z}^3} \psi _{i, n} (x + l) e^{- 2 \pi \textrm{i} k \cdot x} \textrm{d} x \\&= \sum _{l \in \mathbb {Z}^3} \int _{\mathbb {T}^3} \psi _{i, n} (x + l) e^{- 2 \pi \textrm{i} k \cdot x} \textrm{d} x \\&= \sum _{l \in \mathbb {Z}^3} \int _{\mathbb {T}^3 + l} \psi _{i, n} (z) e^{- 2 \pi \textrm{i} k \cdot z} \textrm{d} z \\&= \int _{{\mathbb {R}}^3} \psi _{i, n} (z) e^{- 2 \pi \textrm{i} k \cdot z}\textrm{d} z \\&= (\mathcal {F}_{{\mathbb {R}}^3} \psi _{i, n}) (k) \end{aligned} \end{aligned}$$

where we used $e^{- 2 \pi \textrm{i} k \cdot x} = e^{- 2 \pi \textrm{i} k\cdot (x+ l)}$ $\forall l \in \mathbb {Z}^3$. Hence

38 $$\begin{aligned} \textrm{supp} \mathcal {F}_{\mathbb {T}^3} \psi _{i, n}^\textrm{per} =\mathbb {Z}^3 \cap \textrm{supp} \mathcal {F}_{{\mathbb {R}}^3} \psi _{i, n}. \end{aligned}$$

Furthermore we have

39 $$\begin{aligned} \begin{aligned} (\mathcal {F}_{{\mathbb {R}}^3} \psi _{i, n}) (k)&= (1 + \epsilon _0)^{- \frac{3 n}{2}} (\mathcal {F}_{{\mathbb {R}}^3}\psi _i) ((1 + \epsilon _0)^{- n} k). \end{aligned} \end{aligned}$$

Thus since $\textrm{supp} \mathcal {F}_{{\mathbb {R}}^3} \psi _i \subset B_i \cup -B_i$, it holds

40 $$\begin{aligned} \textrm{supp} \mathcal {F}_{{\mathbb {R}}^3} \psi _{i, n} \subset (1 +\epsilon _0)^n (B_i \cup - B_i) \end{aligned}$$

and

41 $$\begin{aligned} \textrm{supp} \mathcal {F}_{\mathbb {T}^3} \psi _{i, n}^\textrm{per} \subset \mathbb {Z}^3 \cap (1 + \epsilon _0)^n (B_i \cup - B_i). \end{aligned}$$

Finally note that $\psi _{i,n}^\textrm{per}$ is divergence-free. Let $\tilde{\psi }^\textrm{per}_{i, n}$ denote the $L^2$-normalization of $\psi ^\textrm{per}_{i, n}$

$$\begin{aligned} \tilde{\psi }^\textrm{per}_{i, n} (x):= \frac{1}{\Vert \psi _{i,n}^\textrm{per} \Vert _{L^2 (\mathbb {T}^3)}} \psi ^\textrm{per}_{i, n} (x) \end{aligned}$$

and consider the corresponding Cauchy problem

42 $$\begin{aligned} \begin{aligned} \partial _t u&= \varDelta u + C (u, u),\\ u (0, \cdot )&= u_0:= \tilde{\psi }^\textrm{per}_{1, n_0}, \end{aligned} \end{aligned}$$

where

$$\begin{aligned} \begin{aligned} C (u, v)&:= C^\textrm{per} (u, v)\\&:= \sum _{n, i, \mu } \alpha _{i, \mu } (1 + \epsilon _0)^{\frac{5n}{2}} \langle u, \tilde{\psi }_{i_1, n + \mu _1}^\textrm{per} \rangle _{L^2(\mathbb {T}^3)} \langle v, \tilde{\psi }^\textrm{per}_{i_2, n + \mu _2}\rangle _{L^2 (\mathbb {T}^3)} \tilde{\psi }^\textrm{per}_{i_3, n + \mu _3}. \end{aligned} \end{aligned}$$

Analogous to Lemma 1 define

$$\begin{aligned} (\mathcal {F}_{\mathbb {T}^3} u_{i, n} (t)) (k)&:= (\mathcal {F}_{\mathbb {T}^3} u (t)) (k) \mathbbm {1}_{\{ k \in \mathbb {Z}^3 \cap (1 +\epsilon _0)^n (B_i \cup - B_i)\}},\\ X_{i, n} (t)&:= \langle u (t), \tilde{\psi }_{i, n}^\textrm{per}\rangle _{L^2},\\ E_{i, n} (t)&:= \frac{1}{2} \Vert u_{i, n} (t) \Vert ^2_{L^2} . \end{aligned}$$

First we observe the following: it holds

43 $$\begin{aligned} \begin{aligned}&\Vert u_{i,n}(t)\Vert _{H^{\kappa }} \\ {}&= \left\| (\text {Id} - \varDelta )^{\frac{\kappa }{2}}u_{i,n}(t)\right\| _{L^2}\\ {}&= \left( \sum _{k \in \mathbb {Z}^3} \left| \mathcal {F}_{\mathbb {T}^3}\left( (\text {Id} - \varDelta )^{\frac{\kappa }{2}}u_{i,n}(t)\right) \right| ^2\right) ^{\frac{1}{2}}\\ {}&= \left( \sum _{\begin{array}{c} k \in \mathbb {Z}^3 \cap \\ (1+\epsilon _0)^n(B_i \cup - B_i) \end{array}}\left( 1+4\pi ^2|k|^{2n}\right) ^{\kappa }\left| \mathcal {F}_{\mathbb {T}^3}u(t)\right| ^2\right) ^{\frac{1}{2}}\\ {}&\lesssim \left( 1+4\pi ^2(1+\epsilon _0)^{2n}\right) ^{-\frac{\beta }{2}}\left( \sum _{\begin{array}{c} k \in \mathbb {Z}^3 \cap \\ (1+\epsilon _0)^n(B_i \cup - B_i) \end{array}}\left( 1+4\pi ^2|k|^{2n}\right) ^{\kappa +\beta }\left| \mathcal {F}_{\mathbb {T}^3}u(t)\right| ^2\right) ^{\frac{1}{2}}\\ {}&\le \left( 1+4\pi ^2(1+\epsilon _0)^{2n}\right) ^{-\frac{\beta }{2}}\Vert u(t)\Vert _{H^{\kappa +\beta }}\end{aligned} \end{aligned}$$

for any $\kappa , \beta \in {\mathbb {R}}$. Then the blow-up result formulated in Theorem 4 carries over to $\mathbb {T}^3$ as a consequence of the following

#### Theorem 5

Assume that $u:[0,\infty ) \rightarrow H_\textrm{df}^{10}(\mathbb {T}^3)$ is a mild solution to (42), then $(X_{i,n})_{i\in \{1,\ldots ,m\},n\in \mathbb {Z}}$ and $(E_{i,n})_{i\in \{1,\ldots ,m\},n\in \mathbb {Z}}$ satisfy (9)–(16) in Lemma 1.

#### Proof

From (43) we immediately deduce

$$\begin{aligned} \begin{aligned}&\sup _{t\in [0,T]} \sup _{n\in \mathbb {Z}} \sup _{i\in \{1,\ldots ,m\}} (1 + (1 + \epsilon _0)^{10 n}) \sqrt{2 E_{i, n} (t)} \\&= \sup _{t\in [0,T]} \sup _{n\in \mathbb {Z}} \sup _{i\in \{1,\ldots ,m\}} (1 + (1 + \epsilon _0)^{10 n}) \Vert u_{i, n} (t) \Vert _{L^2}\\&\le \sup _{t\in [0,T]} \sup _{n\in \mathbb {Z}} (1 + (1 + \epsilon _0)^{10 n}) (1 + 4 \pi ^2 (1 +\epsilon _0)^{2 n})^{- 5} \Vert u (t) \Vert _{H^{10}}\\&\le \Vert u\Vert _{C_t^0 H_x^{10}} \end{aligned} \end{aligned}$$

as well as

$$\begin{aligned} \sup _{t\in [0,T]} \sup _{n\in \mathbb {Z}} \sup _{i\in \{1,\ldots ,m\}} (1 + (1 + \epsilon _0)^{10 n}) | X_{i, n} (t) | \le \sup _{t,n,i} (1 + (1 + \epsilon _0)^{10 n}) \Vert u_{i, n} (t) \Vert _{L^2} \end{aligned}$$

which gives (9) by $u \in C_t^0H_x^{10}$.

Analysing the time evolution of the above quantities we obtain for $X_{i, n}$

$$\begin{aligned} \partial _t X_{i, n} (t) = \langle \varDelta u (t), \tilde{\psi }_{i,n}^\textrm{per} \rangle _{L^2} + \langle C (u (t), u (t)),\tilde{\psi }_{i, n}^\textrm{per} \rangle _{L^2}. \end{aligned}$$

Using (41), the first summand is of the form

$$\begin{aligned} \begin{aligned} \langle \varDelta u (t), \tilde{\psi }_{i, n}^\textrm{per} \rangle _{L^2}&= \sum _{k \in \mathbb {Z}^3} (\mathcal {F}_{\mathbb {T}^3} (\varDelta u(t))) (k) \cdot (\mathcal {F}_{\mathbb {T}^3} \tilde{\psi }_{i,n}^\textrm{per}) (k)\\&= - 4 \pi ^2 \sum _{k \in \mathbb {Z}^3} | k |^2 (\mathcal {F}_{\mathbb {T}^3} u(t)) (k)\cdot (\mathcal {F}_{\mathbb {T}^3} \tilde{\psi }_{i, n}^\textrm{per}) (k)\\&=- 4 \pi ^2 \sum _{k \in \mathbb {Z}^3\cap (1 +\epsilon _0)^n (B_i \cup - B_i)} | k |^2 (\mathcal {F}_{\mathbb {T}^3} u (t)) (k) \cdot (\mathcal {F}_{\mathbb {T}^3} \tilde{\psi }_{i, n}^\textrm{per}) (k)\\&= - 4 \pi ^2 \sum _{k \in \mathbb {Z}^3} | k |^2 (\mathcal {F}_{\mathbb {T}^3} u_{i, n} (t)) (k)\cdot (\mathcal {F}_{\mathbb {T}^3}\tilde{\psi }_{i, n}^\textrm{per}) (k)\\&= \langle \varDelta u_{i, n} (t), \tilde{\psi }_{i, n}^\textrm{per}\rangle _{L^2}. \end{aligned} \end{aligned}$$

Similarly and since

$$\begin{aligned} \begin{aligned}&(\mathcal {F}_{\mathbb {T}^3} C (u (t), u (t))) (k) \\&= \sum _{n, i, \mu }\alpha _{i, \mu } (1 + \epsilon _0)^{\frac{5 n}{2}} \langle u (t), \tilde{\psi }_{i_1, n + \mu _1}^\textrm{per}\rangle _{L^2} \langle u (t), \tilde{\psi }_{i_2, n + \mu _2}^\textrm{per} \rangle _{L^2} (\mathcal {F}_{\mathbb {T}^3} \tilde{\psi }_{i_3, n + \mu _3}^\textrm{per}) (k) \end{aligned} \end{aligned}$$

we obtain

$$\begin{aligned} \begin{aligned}&\langle C (u (t), u (t)), \tilde{\psi }_{i, n}^\textrm{per} \rangle _{L^2} \\&= \sum _{k \in \mathbb {Z}^3} \sum _{\tilde{n}, j, \mu }\alpha _{j, \mu } (1 + \epsilon _0)^{\frac{5 \tilde{n}}{2}}\langle u (t), \tilde{\psi }_{j_1, \tilde{n} +\mu _1}^\textrm{per} \rangle _{L^2} \langle u (t), \tilde{\psi }_{j_2, \tilde{n} + \mu _2}^\textrm{per} \rangle _{L^2}\\& (\mathcal {F}_{\mathbb {T}^3} \tilde{\psi }_{j_3,\tilde{n} + \mu _3}^\textrm{per}) (k) \cdot (\mathcal {F}_{\mathbb {T}^3} \tilde{\psi }_{i,n}^\textrm{per}) (k)\\&=\sum _{k \in \mathbb {Z}^3} \sum _{i_1, i_2, \mu } \alpha _{i_1, i_2, i,\mu } (1 + \epsilon _0)^{\frac{5 (n - \mu _3)}{2}}\\& \langle u (t), \tilde{\psi }_{i_1, n - \mu _3 +\mu _1}^\textrm{per} \rangle _{L^2} \langle u (t), \tilde{\psi }_{i_2, n - \mu _3 + \mu _2}^\textrm{per} \rangle _{L^2} \left| (\mathcal {F}_{\mathbb {T}^3} \tilde{\psi }_{i,n}^\textrm{per}) (k)\right| ^2\\&=\sum _{i_1, i_2, \mu } \alpha _{i_1, i_2, i, \mu } (1 +\epsilon _0)^{\frac{5 (n - \mu _3)}{2}} X_{i_1, n - \mu _3 + \mu _1} (t) X_{i_2,n - \mu _3 + \mu _2} (t) \end{aligned} \end{aligned}$$

which in total yields

$$\begin{aligned} \begin{aligned} \partial _t X_{i, n} (t)&= \langle \varDelta u_{i, n} (t), \tilde{\psi }_{i,n}^\textrm{per} \rangle _{L^2}\\& + \sum _{i_1, i_2, \mu } \alpha _{i_1, i_2, i, \mu } (1 +\epsilon _0)^{\frac{5 (n - \mu _3)}{2}} X_{i_1, n - \mu _3 + \mu _1} (t) X_{i_2,n - \mu _3 + \mu _2} (t). \end{aligned} \end{aligned}$$

Furthermore by (43) it holds

$$\begin{aligned} \langle \varDelta u_{i, n}(t), \tilde{\psi }_{i, n}^\textrm{per} \rangle _{L^2} \in O\left( (1 + \epsilon _0)^{2 n}\sqrt{E_{i, n}(t)} \right) \end{aligned}$$

which yields (13). Additionally we have

$$\begin{aligned} \begin{aligned} X_{i, n} (0)&= \langle u (0), \tilde{\psi }_{i, n}^\textrm{per} \rangle _{L^2} = \langle u_0, \tilde{\psi }_{i, n}^\textrm{per} \rangle _{L^2} = \langle \tilde{\psi }_{1, n_0}^\textrm{per}, \tilde{\psi }_{i, n}^\textrm{per}\rangle _{L^2}\\&=\mathbbm {1}_{\{(i, n) = (1, n_0)\}}. \end{aligned} \end{aligned}$$

For the local energy we obtain by a similar analysis as for $X_{i, n}$:

$$\begin{aligned} \begin{aligned}&\partial _t E_{i, n} (t) \\&= \sum _{k \in \mathbb {Z}^3}(\mathcal {F}_{\mathbb {T}^3} (\varDelta u_{i, n} (t))) (k)\cdot (\mathcal {F}_{\mathbb {T}^3} u_{i, n} (t)) (k)\\& + \sum _{i_1, i_2, \mu } \alpha _{i_1, i_2, i, \mu } (1 +\epsilon _0)^{\frac{5 (n - \mu _3)}{2}} X_{i_1, n - \mu _3 + \mu _1} (t) X_{i_2,n - \mu _3 + \mu _2} (t)\\& (\mathcal {F}_{\mathbb {T}^3} \tilde{\psi }_{i, n}^\textrm{per})(k)\cdot (\mathcal {F}_{\mathbb {T}^3} u_{i, n} (t)) (k)\\&=\langle \varDelta u_{i, n} (t), u_{i, n} (t) \rangle _{L^2}\\&+ \sum _{i_1, i_2, \mu } \alpha _{i_1, i_2, i, \mu } (1 +\epsilon _0)^{\frac{5 (n - \mu _3)}{2}} X_{i_1, n - \mu _3 + \mu _1} (t) X_{i_2,n - \mu _3 + \mu _2} (t) X_{i, n} (t). \end{aligned} \end{aligned}$$

The rest of the proof is analogous to the proof of Lemma 1 in [23]. $\square$

### Local Well-Posedness

Observe that the analysis in [8] requires the existence of unique local solutions both to (25) as well as (28) and (33) (cf. Remark 1.3 (i) in [8]). Consider systems of the form

44 $$\begin{aligned} \partial _t u = \varDelta u + F(u) \end{aligned}$$

where

45 $$\begin{aligned} \begin{aligned} F(u)&:= C (u, u) \\&= \sum _{n, i, \mu } \alpha _{i, \mu } (1 + \epsilon _0)^{\frac{5n}{2}} \langle u, \tilde{\psi }_{i_1, n + \mu _1}^\textrm{per} \rangle _{L^2(\mathbb {T}^3)} \langle u, \tilde{\psi }^\textrm{per}_{i_2, n + \mu _2}\rangle _{L^2 (\mathbb {T}^3)} \tilde{\psi }^\textrm{per}_{i_3, n + \mu _3}. \end{aligned} \end{aligned}$$

Recall that in Tao’s model, the coefficients $\alpha _{i,\mu }$ are chosen in such a way that the cancellation property

46 $$\begin{aligned} \langle F(u), u \rangle _{L^2} = 0 \end{aligned}$$

holds for all $u \in H_\textrm{df}^{10}$. Thus if $u \in H_\textrm{df}^{10}$, then we easily deduce

47 $$\begin{aligned} \partial _t \Vert u(t)\Vert _{L^2}^2 = -\Vert \nabla u(t)\Vert _{L^2}^2 \end{aligned}$$

and hence the energy equality

48 $$\begin{aligned} \sup _{t \in [0,T]} \Vert u(t)\Vert _{L^2}^2 + \int _0^T\Vert \nabla u(t)\Vert _{L^2}^2\textrm{d}t = \Vert u_0\Vert _{L^2}^2 \end{aligned}$$

for $T \in [0,\infty ]$. Thus let us define a weak solution to (44) in the following way:

#### Definition 2

A vector field $u \in L^{\infty }(0,T;L^2_\textrm{df}(\mathbb {T}^3)) \cap L^2(0,T; H^1_\textrm{df}(\mathbb {T}^3))$ is called a weak solution to (44) if

49 $$\begin{aligned} \begin{aligned}&-\int _0^T \int _{\mathbb {T}^3} \langle u, \partial _t \phi \rangle \textrm{d}x \textrm{d}t - \int _0^T \int _{\mathbb {T}^3} \langle F(u), \phi \rangle \textrm{d}x \textrm{d}t +\int _0^T\int _{\mathbb {T}^3} \langle \nabla u, \nabla \phi \rangle \textrm{d}x \textrm{d}t\\&= \int _{\mathbb {T}^3} \langle u_0, \phi (0) \textrm{d}x \end{aligned} \end{aligned}$$

for any divergence-free test function $\phi \in C^{\infty }_c([0,T)\times \mathbb {T}^3)$.

In this section, we shall discuss whether there exist unique weak solutions to (44). In general, local existence and uniqueness are guaranteed by the hypotheses (H1)–(H3) roughly as follows: first considering a Galerkin approximation on a finite-dimensional subspace, (H1) and (H3) provide that locally, corresponding solutions exist and are unique. Moreover by (H2), they satisfy an energy inequality, and with the help of (H1) again we may pass to the limit to recover local unique solutions for the original system.

#### Violation of Hypotheses

It turns out, however, that for $u \in L^{\infty }(0,T;L^2_\textrm{df}(\mathbb {T}^3)) \cap L^2(0,T; H^1_\textrm{df}(\mathbb {T}^3))$ neither of the hypotheses is satisfied:

##### Theorem 6

The operator *F* as defined in (45) does not satisfy (H1)–(H3).

##### Proof

In attempting to prove the hypotheses, the procedure is as follows: in order to estimate terms of the form $|\langle F(u), \phi \rangle |$, we need to first justify the interchange of integration and summation over $n \in \mathbb {Z}$, i.e. that

$$\begin{aligned} \sum _{n, i, \mu }\left| \alpha _{i, \mu } (1 + \epsilon _0)^{\frac{5n}{2}} \langle u, \tilde{\psi }_{i_1, n + \mu _1}^\textrm{per} \rangle _{L^2} \langle u, \tilde{\psi }^\textrm{per}_{i_2, n + \mu _2}\rangle _{L^2} \langle \tilde{\psi }^\textrm{per}_{i_3, n + \mu _3}, \phi \rangle \right| \end{aligned}$$

is well-defined. We consider the sums over $n<0$ and $n \ge 0$ separately: in the former case since the factor $(1 + \epsilon _0)^{\frac{5n}{2}}$ is already summable for $n<0$, we may crudely estimate terms of the form $\langle v, \tilde{\psi }^\textrm{per}_{i, n}\rangle _{L^2}$ by $\Vert v\Vert _{H^{\kappa }}$ for any $\kappa \ge 0$ using that the functions $\tilde{\psi }^\textrm{per}_{i, n}$ are $L^2$-normalized. In the case of $n \in {\mathbb {N}}_0$, instead estimate via the observation (43) to compensate the in this case diverging factor $(1 + \epsilon _0)^{\frac{5n}{2}}$.

Violation of (H1):

Let $u_1, u_2 \in H^{1-\eta }(\mathbb {T}^3)$ and $\phi \in H^1(\mathbb {T}^3)$, then in view of $|\langle F(u_1) - F(u_2), \phi \rangle |$ we estimate the summands in

$$\begin{aligned} \begin{aligned}&\sum _{n,i,\mu } \left| \alpha _{i,\mu } (1+\epsilon _0)^{\frac{5n}{2}}\langle \tilde{\psi }^\text {per}_{i_3, n+\mu _3}, \phi \rangle \right. \\ {}&\qquad \left. \left( \langle u_1 - u_2, \tilde{\psi }^\text {per}_{i_1, n+\mu _1}\rangle _{L^2} \langle u_1, \tilde{\psi }^\text {per}_{i_2, n+ \mu _2}\rangle _{L^2} + \langle u_2, \tilde{\psi }^\text {per}_{i_1, n+\mu _1}\rangle _{L^2} \langle u_1 - u_2, \tilde{\psi }^\text {per}_{i_2, n+\mu _2}\rangle _{L^2} \right) \right| .\end{aligned} \end{aligned}$$

For $n \in {\mathbb {N}}_0$, we estimate

50 $$\begin{aligned} \begin{aligned}&\left| (1 + \epsilon _0)^{\frac{5n}{2}} \langle u_1 - u_2, \tilde{\psi }_{i_1, n + \mu _1}^\textrm{per} \rangle _{L^2} \langle u_1, \tilde{\psi }^\textrm{per}_{i_2, n + \mu _2}\rangle _{L^2} \langle \tilde{\psi }^\textrm{per}_{i_3, n + \mu _3}, \phi \rangle \right| \\&\lesssim \left( 1+ 4\pi ^2(1+\epsilon _0)^{2n}\right) ^{\frac{1}{4}-\eta }\Vert u_1 -u_2\Vert _{H^{1-\eta }}\Vert u_1\Vert _{H^{1-\eta }}\Vert \phi \Vert _{H^1} \end{aligned} \end{aligned}$$

which is summable for $\eta < \frac{1}{4}$. However in view of the second claim on $\Vert F(u)\Vert _{H^{-1}(\mathbb {T}^3)}$, we obtain

51 $$\begin{aligned} \begin{aligned}&\left| (1 + \epsilon _0)^{\frac{5n}{2}} \langle u, \tilde{\psi }_{i_1, n + \mu _1}^\textrm{per} \rangle _{L^2} \langle u, \tilde{\psi }^\textrm{per}_{i_2, n + \mu _2}\rangle _{L^2} \langle \tilde{\psi }^\textrm{per}_{i_3, n + \mu _3}, \phi \rangle \right| \\&\lesssim \left( 1+ 4\pi ^2(1+\epsilon _0)^{2n}\right) ^{\frac{1}{4}}\Vert u\Vert _{L^2}\Vert u\Vert _{H^1}\Vert \phi \Vert _{H^1} \end{aligned} \end{aligned}$$

which is not summable over $n \in {\mathbb {N}}_0$.

Violation of (H2):

Observe that even the more general form (H2’) in Remark 2, (3), is not satisfied: let $\alpha , \beta , \gamma \in [0,1]$, then interpolation gives an estimate of the form

$$\begin{aligned} \left( 1+ 4\pi ^2(1+\epsilon _0)^{2n}\right) ^{\frac{5}{4}-\frac{1}{2}(\alpha + \beta +\gamma )}\Vert u\Vert ^{\frac{3-(\alpha + \beta + \gamma )}{1+\delta }}_{H^{-\delta }}\Vert u\Vert ^{\frac{3\delta + \alpha + \beta + \gamma }{1+\delta }}_{H^1}\Vert \phi \Vert _{H^1} \end{aligned}$$

for which (H2’) requires

$$\begin{aligned} \frac{3\delta +\alpha + \beta + \gamma }{1+\delta } \in (0,2) \end{aligned}$$

whereas for summability we need $\alpha + \beta +\gamma > \frac{5}{2}$ yielding

$$\begin{aligned} \frac{3\delta + \alpha + \beta + \gamma }{1+\delta }> 3 - \frac{1}{2(1+\delta )} > 2. \end{aligned}$$

Violation of (H3):

We show that also here the more general form (H3’) (see Remark 2, (4)) is violated: let $\gamma \in [0,1]$, then we first estimate

52 $$\begin{aligned} |\langle u_1 - u_2, F(u_1) - F(u_2)\rangle | \le \Vert u_1 -u_2 \Vert _{H^{\gamma }}\Vert F(u_1) - F(u_2) \Vert _{H^{-\gamma }}. \end{aligned}$$

Let $\alpha , \beta \in [0,1]$, then similar to our analysis for (H1) we estimate via interpolation

53 $$\begin{aligned} \begin{aligned}&\left| (1 + \epsilon _0)^{\frac{5n}{2}} \langle u_1 - u_2, \tilde{\psi }_{i_1, n + \mu _1}^\text {per} \rangle _{L^2} \langle u_1, \tilde{\psi }^\text {per}_{i_2, n + \mu _2}\rangle _{L^2} \langle \tilde{\psi }^\text {per}_{i_3, n + \mu _3}, \phi \rangle \right| \\ {}&\lesssim \left( 1+ 4\pi ^2(1+\epsilon _0)^{2n}\right) ^{\frac{5}{4}-\frac{1}{2}(\alpha + \beta +\gamma )}\\ {}&\quad \Vert u_1 -u_2\Vert ^{1-\alpha }_{L^2}\Vert u_1 -u_2\Vert ^{\alpha }_{H^1}\Vert u_1\Vert ^{1-\beta }_{L^2}\Vert u_1\Vert ^{\beta }_{H^1}\Vert \phi \Vert _{H^1} \end{aligned} \end{aligned}$$

hence summability requires again $\alpha + \beta +\gamma > \frac{5}{2}$. Together with (52) we obtain a total estimate of the form

54 $$\begin{aligned} \begin{aligned}&|\langle u_1 - u_2, F(u_1) - F(u_2)\rangle |\\&\lesssim \Vert u_1 -u_2\Vert ^{2-(\alpha +\gamma )}_{L^2}\Vert u_1 -u_2\Vert ^{\alpha +\gamma }_{H^1}\left( \Vert u_1\Vert ^{1-\beta }_{L^2}\Vert u_1\Vert ^{\beta }_{H^1} + \Vert u_2\Vert ^{1-\beta }_{L^2}\Vert u_2\Vert ^{\beta }_{H^1}\right) . \end{aligned} \end{aligned}$$

Hypotheses (H3’) hence requires in particular that

$$\begin{aligned} (\alpha +\gamma ) + \beta _2 \le 2 \end{aligned}$$

which however violates the above summability condition

$$\begin{aligned} 0> \frac{5}{4}-\frac{1}{2}(\alpha + \beta +\gamma ) \ge \frac{1}{4}. \end{aligned}$$

$$\square $$

The take-away message from this proof is that though at first sight the cascade operators are of seemingly simple structure, it is the factor $(1 + \epsilon _0)^{\frac{5n}{2}}$ that dictates whether one may deduce the desired estimates. Note that this factor encodes the relation of the cascade operator to the Euler bilinear operator *B* (at least in a dyadic framework as in [14]) and mimics its scaling behaviour. Furthermore our analysis works irrespective of the precise form of the coefficients $\alpha _{i,\mu }$ whereas in [23] these parameters are carefully chosen so as to facilitate the blow-up.

##### Remark 3

A similar behaviour can be observed in the case of standard NSE: consider

55 $$\begin{aligned} \partial _t u = \varDelta u + F(u) \end{aligned}$$

with $F(u) = B(u,u)=-\Pi ((u\cdot \nabla )u)$, then we may investigate the hypotheses with the help of [24, Lemma 2.1] stating that

$$\begin{aligned} |\langle B(u,v), w\rangle | \lesssim \Vert u\Vert _{H^{m_1}}\Vert v\Vert _{H^{m_2+1}}\Vert w\Vert _{H^{m_3}} \end{aligned}$$

where

56 $$\begin{aligned} \frac{3}{2} \le m_1 + m_2 + m_3,\quad 0\le m_i \ne \frac{3}{2}, i=1,2,3. \end{aligned}$$

Violation of (H1) follows immediately. For (H2’) observe that by interpolation and using $m_2 =0$ we may estimate

57 $$\begin{aligned} |\langle B(u,u),u\rangle | \le \Vert u\Vert _{H^{-\delta }}^{\frac{2-(m_1+m_3)}{1+\delta }}\Vert u\Vert _{H^1}^{1+\frac{m_1+m_3+2\delta }{1+\delta }} \end{aligned}$$

where (H2’) requires

$$\begin{aligned} 1+\frac{m_1+m_3+2\delta }{1+\delta }< 2 \quad \Rightarrow \quad m_1 + m_3 < 1 - \delta \end{aligned}$$

which is in conflict with the requirement (56). In the case of (H3’), for two divergence-free vector fields $u_1, u_2$ we use the identity

58 $$\begin{aligned} |\langle u_1-u_2, F(u_1)- F(u_2) \rangle | = |\langle u_1-u_2, (u_1\cdot \nabla )(u_1-u_2) + ((u_1-u_2)\cdot \nabla )u_2 \rangle |. \end{aligned}$$

Then by [24, Lemma 2.1] we estimate

59 $$\begin{aligned} |\langle (u_1\cdot \nabla )(u_1-u_2), u_1-u_2 \rangle |\le \Vert u_1\Vert _{H^{m_1}}\Vert u_1-u_2\Vert _{H^{m_2+1}}\Vert u_1-u_2\Vert _{H^{m_3}} \end{aligned}$$

hence we require $m_2 =0$. By interpolation it holds

60 $$\begin{aligned} |\langle (u_1\cdot \nabla )(u_1-u_2), u_1-u_2 \rangle |\le \Vert u_1\Vert _{L^2}^{1-m_1}\Vert u_1\Vert _{H^1}^{m_1}\Vert u_1-u_2\Vert _{L^2}^{1-m_3}\Vert u_1-u_2\Vert _{H^1}^{1+m_3} \end{aligned}$$

where (H3’) requires

$$\begin{aligned} (1+m_3) + m_1 \le 2 \quad \Rightarrow \quad m_1 + m_3 \le 1 \end{aligned}$$

violating (56). One proceeds similarly for the second summand.

#### Order of Well-Posed Derivatives

Denote $v:= (\textrm{Id} - \varDelta )^{\rho } u$, then *v* satisfies

61 $$\begin{aligned} \partial _t v = \varDelta v + F_{\rho }(v) \end{aligned}$$

where

62 $$\begin{aligned} F_{\rho }(v):= (\textrm{Id}-\varDelta )^{\rho } F(u). \end{aligned}$$

This section shall discuss the minimal threshold value of $\rho >0$ for which $F_{\rho }$ is well-defined in the sense that

63 $$\begin{aligned} F_{\rho }(v) = \sum _{n, i, \mu }\alpha _{i,\mu } (1+\epsilon _0)^{\frac{5n}{2}}\langle u, \tilde{\psi }_{i_1, n + \mu _1}^\textrm{per} \rangle _{L^2} \langle u, \tilde{\psi }^\textrm{per}_{i_2, n + \mu _2}\rangle _{L^2} (\textrm{Id}-\varDelta )^{\rho } \tilde{\psi }^\textrm{per}_{i_3, n + \mu _3} \end{aligned}$$

and attains the hypotheses from Sect. 2.2.2. First recall that it holds

64 $$\begin{aligned} \Vert v\Vert _{H^{\gamma }} =\left\| (\textrm{Id} - \varDelta )^{\frac{\gamma }{2}}v\right\| _{L^2}=\left\| (\textrm{Id}-\varDelta )^{\rho + \frac{\gamma }{2}}u\right\| _{L^2} = \Vert u\Vert _{H^{2\rho +\gamma }} \end{aligned}$$

and denote $$v_i= (\textrm{Id}-\varDelta )^{\rho }u_i$$, $$i=1,2$$.

##### Theorem 7

$F_{\rho }$ satisfies (H1), (H2’) and (H3’) if $\rho > \frac{1}{8}$.

##### Proof

In case of (H1), claim 2, we estimate for $n \in {\mathbb {N}}_0$

65 $$\begin{aligned} \begin{aligned}&\left| (1+\epsilon _0)^{\frac{5n}{2}}\langle u,\tilde{\psi }^\textrm{per}_{i_1, n+\mu _1}\rangle _{L^2} \langle u,\tilde{\psi }^\textrm{per}_{i_2, n+\mu _2}\rangle _{L^2} \langle (\textrm{Id}-\varDelta )^{\rho }\tilde{\psi }^\textrm{per}_{i_3, n+\mu _3}, \phi \rangle _{L^2}\right| \\&\lesssim \left( 1+4\pi ^2(1+\epsilon _0)^{2n}\right) ^{\frac{5}{4}-(2\rho +1)}\Vert u\Vert _{H^{2\rho }}\Vert u\Vert _{H^{2\rho +1}}\Vert \phi \Vert _{H^1} \end{aligned} \end{aligned}$$

which is summable if

$$\begin{aligned} \rho > \frac{1}{8}. \end{aligned}$$

For (H2’) we use interpolation as in the proof of Theorem 6 to obtain the estimate

66 $$\begin{aligned} \begin{aligned}&\left| (1 + \epsilon _0)^{\frac{5n}{2}} \langle u, \tilde{\psi }_{i_1, n + \mu _1}^\textrm{per} \rangle _{L^2} \langle u, \tilde{\psi }^\textrm{per}_{i_2, n + \mu _2}\rangle _{L^2 } \langle (\textrm{Id}-\varDelta )^{\rho } \tilde{\psi }^\textrm{per}_{i_3, n + \mu _3}, u \rangle \right| \\&\lesssim \left( 1+ 4\pi ^2(1+\epsilon _0)^{2n}\right) ^{\frac{5}{4}-\frac{1}{2}(\alpha + \beta +\gamma )}\Vert u\Vert _{H^{\alpha } }\Vert u\Vert _{H^{\beta }}\Vert (\textrm{Id}-\varDelta )^{\rho }u\Vert _{H^{\gamma } }\\&\le \left( 1+ 4\pi ^2(1+\epsilon _0)^{2n}\right) ^{\frac{5}{4}-\frac{1}{2}(\alpha + \beta +\gamma )}\Vert v\Vert ^{\frac{3-(\alpha + \beta + \gamma -4\rho )}{1+\delta }}_{H^{-\delta }}\Vert u\Vert ^{\frac{3\delta + \alpha + \beta + \gamma -4\rho }{1+\delta }}_{H^1} \end{aligned} \end{aligned}$$

which is summable if $\alpha + \beta +\gamma > \frac{5}{2}$ and satisfies the requirements of (H2’) if

$$\begin{aligned} \rho> \frac{1}{4}\left( \delta + \alpha + \beta + \gamma -2\right)> \frac{1}{4}\left( \delta + \frac{1}{2}\right) > \frac{1}{8}. \end{aligned}$$

Finally for (H3’), let $\gamma \in [0,1]$, then we first estimate

67 $$\begin{aligned} |\langle v_1 - v_2, F_{\rho }(v_1) - F_{\rho }(v_2)\rangle | \le \Vert v_1 -v_2 \Vert _{H^{\gamma }}\Vert F_{\rho }(v_1) - F_{\rho }(v_2) \Vert _{H^{-\gamma }}. \end{aligned}$$

We continue as in the proof of Theorem 6: in the case of $n \in {\mathbb {N}}_0$, let $\alpha$ and $\beta$ be such that $\alpha - 2 \rho , \beta - 2\rho \in [0,1]$. Then via interpolation we obtain for $\phi \in H^{\gamma }(\mathbb {T}^3)$

68 $$\begin{aligned} \begin{aligned}&\left| (1+\epsilon _0)^{\frac{5n}{2}}\langle u_1 - u_2, \tilde{\psi }^\text {per}_{i_1, n+\mu _1}\rangle _{L^2} \langle u_1, \tilde{\psi }^\text {per}_{i_2, n+ \mu _2}\rangle _{L^2}\langle (\text {Id}-\varDelta )^{\rho }\tilde{\psi }^\text {per}_{i_3, n+\mu _3}, \phi \rangle \right| \\ {}&\lesssim \left( 1+4\pi ^2(1+\epsilon _0)^{2n}\right) ^{\frac{5}{4}-\frac{1}{2}(\alpha + \beta +\gamma -2\rho )}\Vert u_1 - u_2 \Vert _{H^{\alpha }} \Vert u_1\Vert _{H^{\beta }} \Vert \phi \Vert _{H^{\gamma }}\\ {}&\le \left( 1+4\pi ^2(1+\epsilon _0)^{2n}\right) ^{\frac{5}{4}-\frac{1}{2}(\alpha + \beta +\gamma -2\rho )}\\ {}&\qquad \Vert v_1 - v_2 \Vert ^{1-(\alpha -2\rho )}_{L^2} \Vert v_1-v_2\Vert ^{\alpha -2\rho }_{H^1}\Vert v_1\Vert ^{1-(\beta -2\rho )}_{L^2}\Vert v_1\Vert ^{\beta -2\rho }_{H^1} \Vert \phi \Vert _{H^{\gamma }}\end{aligned} \end{aligned}$$

which is summable if $\alpha + \beta +\gamma > \frac{5}{2}$. Thus we obtain

69 $$\begin{aligned} \begin{aligned}&|\langle v_1 - v_2, F_{\rho }(v_1) - F_{\rho }(v_2)\rangle |\\ {}&\lesssim \Vert v_1 - v_2 \Vert ^{2-(\alpha +\gamma -2\rho )}_{L^2} \Vert v_1-v_2\Vert ^{\alpha +\gamma -2\rho }_{H^1}\\ {}&\quad \left( \Vert v_1\Vert ^{1-(\beta -2\rho )}_{L^2}\Vert v_1\Vert ^{\beta -2\rho }_{H^1} + \Vert v_2\Vert ^{1-(\beta -2\rho )}_{L^2}\Vert v_2\Vert ^{\beta -2\rho }_{H^1}\right) \end{aligned} \end{aligned}$$

which satisfies the requirements in (H3’) if

$$\begin{aligned} 2(1-2\rho ) \ge \alpha + \beta +\gamma> \frac{5}{2} \quad \Rightarrow \quad \rho > \frac{1}{8}. \end{aligned}$$

$$\square $$

##### Remark 4

In case of the standard NSE, using [24, Lemma 2.1] we estimate for (H1), claim 2,

70 $$\begin{aligned} \begin{aligned} |\langle (\textrm{Id}-\varDelta )^{\rho } B(u,u), \phi \rangle |&\le \Vert u\Vert _{H^{m_1}}\Vert u\Vert _{H^{m_2+1}}\Vert (\textrm{Id}-\varDelta )^{\rho }\phi \Vert _{H^{m_3}} \\&= \Vert v\Vert _{H^{m_1-2\rho }}\Vert v\Vert _{H^{m_2+1-2\rho }}\Vert \phi \Vert _{H^{m_3+2\rho }}. \end{aligned} \end{aligned}$$

Hence we require $m_3 = 1-2\rho$. Since claim 2 in (H1) requires for an estimate involving the $L^2(\mathbb {T}^3)$- and the $H^1(\mathbb {T}^3)$-norm, assume $m_1 - 2\rho \in [0,1], m_2+1-2\rho \in [0,1]$. Via interpolation we obtain

$$\begin{aligned} \Vert v\Vert _{H^{m_1-2\rho }}\Vert v\Vert _{H^{m_2+1-2\rho }} \lesssim \Vert v\Vert _{L^2}^{1+4\rho - (m_1+m_2)}\Vert v\Vert _{H^1}^{1-4\rho +m_1 + m_2} \end{aligned}$$

and claim 2 in (H1) requires

$$\begin{aligned} 1-4\rho +m_1 + m_2 = 1 \quad \Rightarrow \quad 4\rho = m_1 + m_2. \end{aligned}$$

Further with the requirement in [24, Lemma 2.1] we obtain

$$\begin{aligned} \frac{3}{2} \le m_1 + m_2 + m_3 = 1 + 2\rho \quad \Rightarrow \quad \frac{1}{4} \le \rho . \end{aligned}$$

This threshold equally holds in the case of (H2’) and (H3’) via a similar analysis.

In terms of regularizability as specified by Theorem 4, we finally need to check whether also (H4) is satisfied:

##### Theorem 8

If $\rho > \frac{1}{4}$, then we obtain (H4) with $\mathcal {K}$ as in Remark 2, (30).

##### Proof

In view of Remark 2, (30), we show that the system (61) preserves the set of mean-zero functions in $L^2({\mathbb {R}}^3)$. More precisely we show that $F_{\rho }$ has zero mean: first observe that

71 $$\begin{aligned} \begin{aligned} \int _{\mathbb {T}^3} \left( (\textrm{Id}-\varDelta )^{\rho } \tilde{\psi }^\textrm{per}_{i_3, n + \mu _3}\right) (x)\textrm{d} x&= \frac{1}{2^3} \mathcal {F}_{\mathbb {T}^3}\left( (\textrm{Id}-\varDelta )^{\rho } \tilde{\psi }^\textrm{per}_{i_3, n + \mu _3}\right) (0)\\&= \frac{1}{2^3} \left( (1+4\pi ^2|\cdot |^2)^{\rho } \mathcal {F}_{\mathbb {T}^3}\tilde{\psi }^\textrm{per}_{i_3, n + \mu _3}\right) (0)\\&= \frac{1}{2^3} \left( \mathcal {F}_{\mathbb {T}^3}\tilde{\psi }^\textrm{per}_{i_3, n + \mu _3}\right) (0) =0 \end{aligned} \end{aligned}$$

since $\mathcal {F}_{\mathbb {T}^3}\tilde{\psi }^\textrm{per}_{i, n}$ are supported away from 0. In order to justify that we may interchange integration and derivatives with summation, further observe that it holds with the help of (43)

72 $$\begin{aligned} \begin{aligned} \int _{\mathbb {T}^3} \left| \left( (\textrm{Id}-\varDelta )^{\rho } \tilde{\psi }^\textrm{per}_{i_3, n + \mu _3}\right) (x)\right| \textrm{d} x&\le \left\| \tilde{\psi }^\textrm{per}_{i_3, n + \mu _3}\right\| _{H^{2\rho }}\\&\lesssim \left( 1 + 4\pi ^2 (1+\epsilon _0)^{2n}\right) ^{\rho }. \end{aligned} \end{aligned}$$

Hence using $u(t) \in H^1(\mathbb {T}^3)$ together with (43) we estimate

73 $$\begin{aligned} \begin{aligned}&(1+\epsilon _0)^{\frac{5n}{2}}|\langle u, \tilde{\psi }_{i_1, n + \mu _1}^\textrm{per} \rangle _{L^2}| |\langle u, \tilde{\psi }^\textrm{per}_{i_2, n + \mu _2}\rangle _{L^2}|\int _{\mathbb {T}^3}\left| \left( (\textrm{Id}-\varDelta )^{\rho } \tilde{\psi }^\textrm{per}_{i_3, n + \mu _3}\right) (x)\right| \textrm{d} x\\&\lesssim \left( 1 + 4\pi ^2 (1+\epsilon _0)^{2n}\right) ^{\frac{1}{4} - \rho }. \end{aligned} \end{aligned}$$

Hence for $\rho > \frac{1}{4}$ we justified

74 $$\begin{aligned} \begin{aligned} \int _{\mathbb {T}^3} F_{\rho }(v)(x)\text {d}x&= \int _{\mathbb {T}^3} \sum _{n, i, \mu }\alpha _{i,\mu } (1+\epsilon _0)^{\frac{5n}{2}}\langle u, \tilde{\psi }_{i_1, n + \mu _1}^\text {per} \rangle _{L^2} \langle u, \tilde{\psi }^\text {per}_{i_2, n + \mu _2}\rangle _{L^2}\\ {}&\left( (\text {Id}-\varDelta )^{\rho } \tilde{\psi }^\text {per}_{i_3, n + \mu _3}\right) (x) \text {d}x\\ {}&= \sum _{n, i, \mu }\alpha _{i,\mu } (1+\epsilon _0)^{\frac{5n}{2}}\langle u, \tilde{\psi }_{i_1, n + \mu _1}^\text {per} \rangle _{L^2} \langle u, \tilde{\psi }^\text {per}_{i_2, n + \mu _2}\rangle _{L^2}\\ {}&\int _{\mathbb {T}^3} \left( (\text {Id}-\varDelta )^{\rho } \tilde{\psi }^\text {per}_{i_3, n + \mu _3}\right) (x)\text {d} x =0. \end{aligned} \end{aligned}$$

$$\square $$

### Regularization of the Periodic Averaged NSE

Recall that in the case of the periodic averaged NSE we consider the system (44) given by

75 $$\begin{aligned} \begin{aligned} \partial _t u&= \varDelta u + C (u, u),\\ u (0, \cdot )&= \tilde{\psi }^\textrm{per}_{1, n_0}, \end{aligned} \end{aligned}$$

and the corresponding blow-up result holds in the setting of $H_\textrm{df}^{10}(\mathbb {T}^3)$. Let

$$\begin{aligned} v = (\textrm{Id} - \varDelta )^5u, \end{aligned}$$

then on the one hand clearly it holds

$$\begin{aligned} \Vert v(t)\Vert _{L^2} = \Vert (\textrm{Id} - \varDelta )^5u(t)\Vert _{L^2} = \Vert u(t)\Vert _{H^{10}}, \end{aligned}$$

and on the other hand (H1)–(H4) hold as seen in the previous section. We may therefore conclude:

#### Theorem 9

For arbitrary large time $T \in (0,\infty )$ and $\nu = \nu (T) >0$ as in (H4) the solution to

76 $$\begin{aligned} \begin{aligned} \textrm{d} v&= (\varDelta v + F_5(v))\textrm{d} t + \frac{\sqrt{C_3 \nu }}{\Vert \theta \Vert _{\ell ^2}}\sum _{k \in \mathbb {Z}_0^3}\sum _{i=1}^{2}\theta _k \Pi ((\sigma _{k,i}\cdot \nabla )v) \circ \textrm{d}W^{k,i},\\ v(0,\cdot )&= (\textrm{Id} - \varDelta )^5\tilde{\psi }^\textrm{per}_{1, n_0}, \end{aligned} \end{aligned}$$

does not blow up in $C([0,T];L^2_\textrm{df}(\mathbb {T}^3))$ with high probability in the sense of Theorem 4. In particular, this implies delay of blow-up for solutions to (75) in $H_\textrm{df}^{10}(\mathbb {T}^3)$.

## Data Availability

This manuscript has no associated data.

## Appendix A: Proof of Theorem 4

The proof closely follows [8] making use of results from [7], thereby using the same or similar notation: let $T, R >0$ be fixed parameters. As in [7] and [8], we first consider the cut-off equation

77 $$\begin{aligned} \begin{aligned} \textrm{d}u&= \left( \varLambda ^{2\alpha }u + g_R(u)F(u)\right) \textrm{d}t + \frac{\sqrt{C_d \nu }}{\Vert \theta \Vert _{\ell ^2}}\sum _{k \in \mathbb {Z}_0^d}\sum _{i=1}^{d-1}\theta _k \Pi ((\sigma _{k,i}\cdot \nabla )u)\circ \textrm{d}W^{k,i},\\ u(0, \cdot )&= u_0, \end{aligned} \end{aligned}$$

where we denote $\varLambda ^{2\alpha }:= (-\varDelta )^{\alpha }$ and $g_R(u):= g_R\left( \Vert u\Vert _{H^{-\delta }}\right) , \delta >0$, for a Lipschitz-continuous cut-off function $g_R:[0,\infty ) \rightarrow [0,1]$ with $g_R(x) =1$ for $x \in [0,R]$, and $g_R(x) =0$ for $x >R+1$. Similarly to [7] we obtain the corresponding Itô-formulation

78 $$\begin{aligned} \begin{aligned} \text {d}u&= \left( \varLambda ^{2\alpha }u + g_R(u)F(u)+S_{\theta }(u)\right) \text {d}t \\ {}&\quad + \frac{\sqrt{C_d \nu }}{\Vert \theta \Vert _{\ell ^2}}\sum _{k \in \mathbb {Z}_0^d}\sum _{i=1}^{d-1}\theta _k \Pi ((\sigma _{k,i}\cdot \nabla )u) \text {d}W^{k,i},\\ u(0, \cdot )&= u_0, \end{aligned} \end{aligned}$$

where

79 $$\begin{aligned} S_{\theta }(u):= \frac{C_d \nu }{\Vert \theta \Vert ^2_{\ell ^2}} \sum _{k \in \mathbb {Z}_0^d}\sum _{i=1}^{d-1}\theta _k^2 \Pi \left( (\sigma _{k,i}\cdot \nabla )\Pi ((\sigma _{-k,i}\cdot \nabla )u)\right) . \end{aligned}$$

### Definition 3

(cf. [7, Definition 1.1] and [8, Definition 3.1]) Let $(\Omega , \mathcal {F}, (\mathcal {F}_t), \mathbb {P})$ be a probability space with a family of Brownian motions $\left\{ W^{k,i} \right\}$ as in Sect. 2.2. Further let $u_0 \in L^2(\mathbb {T}^d)$ be divergence-free. A process *u* with trajectories in $C([0,T],L^2_\textrm{df}(\mathbb {T}^d) \cap L^2(0,T;H^{\alpha }_\textrm{df}(\mathbb {T}^d))$ is a strong solution to (77), if it is $(\mathcal {F}_t)$-adapted and for any $\phi \in H^{\alpha }_\textrm{df}(\mathbb {T}^d)$ $(\langle u(t), \Pi ((\sigma _{k,i}\cdot \nabla )\phi )\rangle _{L^2(\mathbb {T}^d)})_t$ is an $(\mathcal {F}_t)$-continuous semimartingale, and with probability one it holds for all $t \in [0,T]$

80 $$\begin{aligned} \begin{aligned}&\langle u(t), \phi \rangle _{L^2(\mathbb {T}^d)} \\ {}&= \langle u_0, \phi \rangle _{L^2(\mathbb {T}^d)} \\ {}&\quad + \int _0^t \langle -\varLambda ^{2\alpha }u(s)+ g_R(u(s)) F(u(s)) + S_{\theta }(u(s)),\phi \rangle _{L^2(\mathbb {T}^d)}\text {d}s\\ {}&\quad + \frac{\sqrt{C_d \nu }}{\Vert \theta \Vert _{\ell ^2}}\sum _{k \in \mathbb {Z}_0^d}\sum _{i=1}^{d-1}\theta _k \int _0^t\langle u(s), \Pi ((\sigma _{k,i}\cdot \nabla )\phi )\rangle _{L^2(\mathbb {T}^d)} \text {d}W^{k,i}.\end{aligned} \end{aligned}$$

In order to show existence of strong solutions in the sense of the above definition, we follow the standard agenda:

1. Show existence of global weak solutions to
  81 $$\begin{aligned} \begin{aligned} \text {d}u&= \left( -\varLambda ^{2\alpha } u + g_R(u)F(u) + S_{\theta }(u)\right) \text {d}t \\ {}&\quad +\frac{\sqrt{C_d \nu }}{\Vert \theta \Vert _{\ell ^2}}\sum _{k \in \mathbb {Z}_0^d}\sum _{i=1}^{d-1}\theta _k \Pi ((\sigma _{k,i}\cdot \nabla )u) \text {d}W^{k,i}.\end{aligned} \end{aligned}$$
2. Show pathwise uniqueness of weak solutions to (81).
3. Conclude via a Yamada–Watanabe argument.

On (1): For $N \in {\mathbb {N}}$, let $H_N:=\{ \sigma _{k,i}: |k|\le N, i=1,\ldots ,d-1\}$, and for *H* the real subspace of $H_{\mathbb {C}}$ (defined in (18)) denote the corresponding orthogonal projection by $\Pi _N: H \rightarrow H_N$. Consider the Galerkin approximation of (81):

82 $$\begin{aligned} \begin{aligned} \text {d}u_N&= \left( -\varLambda ^{2\alpha } u_N + g_R(u_N)\Pi _NF(u_N) + S_{\theta }(u_N)\right) \text {d}t \\ {}&\quad +\frac{\sqrt{C_d \nu }}{\Vert \theta \Vert _{\ell ^2}}\sum _{k \in \mathbb {Z}_0^d}\sum _{i=1}^{d-1}\theta _k \Pi _N((\sigma _{k,i}\cdot \nabla )u_N) \text {d}W^{k,i}. \end{aligned} \end{aligned}$$

We obtain the following a-priori estimates: via Itô’s formula it holds

83 $$\begin{aligned} \begin{aligned}&\text {d}\Vert u_N(t)\Vert ^2_{L^2} \\ {}&= 2 \left\langle u_N(t), -\varLambda ^{2\alpha }u_N(t) + g_R( u_N(t) ) \Pi _N F(u_N(t)) + S_{\theta }(u_N(t))\right\rangle _{L^2} \text {d}t \\ {}&\quad + 2 \left\langle u_N, \frac{\sqrt{C_d \nu }}{\Vert \theta \Vert _{\ell ^2}}\sum _{k \in \mathbb {Z}_0^d}\sum _{i=1}^{d-1}\theta _k \Pi _N((\sigma _{k,i}\cdot \nabla )u_N(t)) \text {d}W^{k,i}\right\rangle _{L^2}\\ {}&\quad + \frac{C_d \nu }{\Vert \theta \Vert ^2_{\ell ^2}} \sum _{k \in \mathbb {Z}_0^d}\sum _{i=1}^{d-1}\theta _k^2 \left\| \Pi _N((\sigma _{k,i}\cdot \nabla )u_N(t))\right\| ^2_{L^2} \text {d}t.\end{aligned} \end{aligned}$$

Observe that on the one hand since $\sigma _{k,i}$ are divergence-free, it holds

$$\begin{aligned} \langle u_N(t), \Pi _N((\sigma _{k,i}\cdot \nabla )u_N(t))\rangle _{L^2} =0 \end{aligned}$$

hence the martingale part vanishes, and on the other hand as seen in [7] it holds

$$\begin{aligned} 2\langle u_N(t), S_{\theta }(u_N(t))\rangle _{L^2} = - \frac{C_d \nu }{\Vert \theta \Vert ^2_{\ell ^2}} \sum _{k \in \mathbb {Z}_0^d}\sum _{i=1}^{d-1}\theta _k^2 \left\| \Pi ((\sigma _{k,i}\cdot \nabla )u_N(t))\right\| ^2_{L^2} \end{aligned}$$

which in total gives

84 $$\begin{aligned} \frac{\textrm{d}}{\textrm{d}t}\Vert u_N(t)\Vert ^2_{L^2} \le -2 \left\| \varLambda ^{\alpha }u\right\| _{L^2} + 2\langle u_N(t), g_R(u_N(t)) \Pi _N F(u_N(t))\rangle _{L^2}. \end{aligned}$$

Using Remark 2 (3) (for which we assume that $\delta >0$ is small enough such that (H2’) holds) and the analysis in [8] it holds

85 $$\begin{aligned} \begin{aligned}&|\langle u_N(t), g_R(u_N(t)) \Pi _N F(u_N(t))\rangle _{L^2}| \\&\lesssim 1 + \frac{1}{2^{\alpha }} \Vert u_N(t)\Vert _{H^{\alpha }}^2\\&\le 1 + \frac{1}{2}\left( \Vert u_N(t)\Vert _{L^2}^2 + \left\| \varLambda ^{\alpha }u_N(t)\right\| _{L^2}^2\right) , \end{aligned} \end{aligned}$$

hence we obtain a constant *C* such that

86 $$\begin{aligned} \sup _{t\in [0,T]} \Vert u_N(t)\Vert ^2_{L^2} + \int _0^T \left\| \varLambda ^{\alpha }u_N(t)\right\| ^2_{L^2} \textrm{d}t \le C\left( 1+\Vert u_0\Vert ^2_{L^2}\right) . \end{aligned}$$

In the following we consider the case $\alpha >1$ and follow the analysis in [8] (for $\alpha =1$, proceed by using the results in [7]): recall

### Lemma 2

(cf. [8, Lemma 3.3]) For any $\beta , \gamma , \epsilon >0$ and any $p < \infty$ define

$$\begin{aligned} \mathcal {S}^{\gamma , \beta }&:= L^2(0,T;H^{\alpha }(\mathbb {T}^d)) \cap C([0,T];L^2(\mathbb {T}^d)) \cap C^{\gamma }([0,T];H^{\beta }(\mathbb {T}^d)),\\ \mathcal {X}^{\epsilon , p}&:= L^2(0,T;H^{\alpha - \epsilon }(\mathbb {T}^d)) \cap L^p(0,T;L^2(\mathbb {T}^d)) \cap C([0,T]; H^{-\epsilon }(\mathbb {T}^d)), \end{aligned}$$

then the embedding $\mathcal {S}^{\gamma , \beta } \hookrightarrow \mathcal {X}^{\epsilon , p}$ is compact and for any finite $K\ge 0$ the set

$$\begin{aligned} \mathcal {X}_K:= \left\{ f \in \mathcal {X}^{\epsilon , p}: \sup _{t \in [0,T]} \Vert f(t)\Vert _{L^2(\mathbb {T}^d)} + \Vert f\Vert _{L^2H^{\alpha }(\mathbb {T}^d)} \le K\right\} \end{aligned}$$

is closed in $\mathcal {X}^{\epsilon , p}$ and hence a Polish space with metric inherited from $\mathcal {X}^{\epsilon , p}$.

Hence we proceed to verify that there exist $p>1, \beta , \gamma >0$ such that

87 $$\begin{aligned} \sup _{N \in {\mathbb {N}}} {\mathbb {E}}\left[ \left( \Vert u_N\Vert _{L^2H^{\alpha }} + \Vert u_N\Vert _{L^{\infty }L^2} + \Vert u_N\Vert _{C^{\gamma }H^{-\beta }}\right) ^p\right] < \infty . \end{aligned}$$

Denote

$$\begin{aligned} M_N(t):= \frac{\sqrt{C_d \nu }}{\Vert \theta \Vert _{\ell ^2}} \int _0^t \sum _{k \in \mathbb {Z}_0^d}\sum _{i=1}^{d-1}\theta _k \Pi _N((\sigma _{k,i}\cdot \nabla )u_N(s)) \textrm{d}W^{k,i}_s, \end{aligned}$$

then for any $t,s \in [0,T]$ we deduce from a similar analysis as in [8] that

88 $$\begin{aligned} {\mathbb {E}}\left[ \Vert M_N(t) - M_N(s)\Vert _{H^{-\beta }}^{2p}\right] \lesssim \Vert \theta \Vert _{\ell ^{\infty }}^{2p} \left( 1+\Vert u_0\Vert _{L^2}^2\right) ^p |t-s|^p. \end{aligned}$$

Further, by assumption on *F* it holds for

$$\begin{aligned} v_N(t):= \int _0^t -\varLambda ^{2\alpha } u_N(s) + g_R(u_N(s))\Pi _N F(u_N(s)) \textrm{d}s \end{aligned}$$

that

89 $$\begin{aligned} \begin{aligned} \Vert v_N\Vert _{C^{\frac{1}{2}}H^{-\alpha }}&\le \Vert v_N\Vert _{W^{1,2}H^{-\alpha }} \\&\le \left( 1 + \Vert u_N\Vert _{L^{\infty }L^2}^{\beta _1}\right) \left( 1 + \Vert u_N\Vert _{L^2H^{\alpha }}\right) . \end{aligned} \end{aligned}$$

It thus remains to estimate

$$\begin{aligned} \left\| \int _0^{\cdot } S_{\theta }(u_N(s))\textrm{d}s\right\| _{W^{1,2}H^{-\alpha }}: \end{aligned}$$

Observe from [7] that it holds for any $t,s \in [0,T]$ and $l \in \mathbb {Z}_0^d, j \in \{1,\ldots ,d-1\}$

90 $$\begin{aligned} \begin{aligned} \left| \left\langle \int _s^t S_{\theta }(u_N(r)) \textrm{d}r, \sigma _{l,j}\right\rangle _{L^2}\right|&= \left| \int _s^t \langle u_N(r)), S_{\theta }(\sigma _{l,j})\rangle _{L^2}\textrm{d}r \right| \\&\lesssim \Vert u_N\Vert _{L^{\infty }L^2} |l|^2|t-s| \end{aligned} \end{aligned}$$

and hence

91 $$\begin{aligned} \begin{aligned} \left\| \int _s^t S_{\theta }(u_N(r)) \textrm{d}r \right\| _{H^{-\alpha }}^2&= \sum _{l,j} \frac{\left| \left\langle \int _s^t S_{\theta }(u_N(r)) \textrm{d}r, \sigma _{l,j}\right\rangle _{L^2}\right| ^2}{|l|^{-2\alpha }}\\&\lesssim \Vert u_N\Vert _{L^{\infty }L^2}^2 |t-s|^2\sum _l |l|^{2(1-\alpha )} \end{aligned} \end{aligned}$$

where the series converges for $\alpha >1$. In total this gives

92 $$\begin{aligned} \left\| \int _0^{\cdot } S_{\theta }(u_N(s))\textrm{d}s\right\| _{C^{\frac{1}{2}}H^{-\alpha }} \lesssim \Vert u_N\Vert _{L^{\infty }L^2} \end{aligned}$$

and concludes the proof of (87). Thus we obtain existence of weak solutions as in [8] (for the reader’s convenience we repeat here the core arguments): denote $\mu _N:= \textrm{Law}(u_N)$, then by Lemma 2 and Prokhorov’s theorem, the family $\{\mu _N\}_N$ is tight in $\mathcal {X}^{\epsilon , p}$ and we can find *K* large enough such that $\{\mu _N\}_N$ are supported on $\mathcal {X}_K$ and thus tight therein as well. Existence of weak solutions then follows from Skorokhod’s representation theorem: for $W:=(W^{k,i})_{k,i}$ denote $P_N:= \textrm{Law}(u_N, W)$, then $\{P_N\}_N$ is tight in $\mathcal {X}_K \times C\left( [0,T], \mathbb {C}^{\mathbb {Z}_0^2}\right)$. Hence we can find another probability space $(\tilde{\Omega }, \tilde{\mathcal {F}}, (\tilde{\mathcal {F}}_t), \tilde{\mathbb {P}})$ and corresponding $(\tilde{u}_N, \tilde{W}_N)$ such that $(\tilde{u}_N, \tilde{W}_N)$ converge to $(\tilde{u}, \tilde{W})$ $\tilde{\mathbb {P}}$-almost surely in $\mathcal {X}_K \times C\left( [0,T], \mathbb {C}^{\mathbb {Z}_0^2}\right)$ and $\textrm{Law}(\tilde{u}_N, \tilde{W}_N) = P_N$. Furthermore $\tilde{u}$ solves (77) with $\tilde{W}$ by $\tilde{\mathbb {P}}$-almost sure convergence where the nonlinear part converges due to continuity of *F* on $\mathcal {X}_K$ (see Lemma 3.4, [8]).

On (2): For pathwise uniqueness assume that on some probability space $(\Omega , \mathcal {F}, (\mathcal {F}_t), \mathbb {P})$ there exist two weak solutions $u_1, u_2$ to (78) with the same *W* and same initial condition $u_0$. Let $\tilde{u}:=u_1 - u_2$, then for $\phi \in C^{\infty }(\mathbb {T}^d)$ it holds

93 $$\begin{aligned} \begin{aligned}&\langle \tilde{u}(t), \phi \rangle _{L^2} \\ {}&= \int _0^t \langle - \varLambda ^{2\alpha }\tilde{u}(s) +\left( g_R(u_1(s))F(u_1(s))-g_R(u_2(s))F(u_2(s))\right) + S_{\theta }(\tilde{u}(s)), \phi \rangle _{L^2} \text {d}s\\ {}&\quad + \frac{\sqrt{C_d \nu }}{\Vert \theta \Vert _{\ell ^2}} \sum _{k \in \mathbb {Z}_0^d}\sum _{i=1}^{d-1}\theta _k \int _0^t\langle \Pi ((\sigma _{k,i}\cdot \nabla )\tilde{u}(s)), \phi \rangle _{L^2} \text {d}W^{k,i}_s.\end{aligned} \end{aligned}$$

By Itô’s formula we obtain as before

94 $$\begin{aligned} \begin{aligned}&\frac{\textrm{d}}{\textrm{d}t} \Vert \tilde{u}(t)\Vert _{L^2}^2 \\&= -2\Vert \varLambda ^{\alpha }\tilde{u}(t)\Vert _{L^2}^2 + \langle g_R(u_1(t))F(u_1(t))-g_R(u_2(t))F(u_2(t)), \tilde{u}(t)\rangle _{L^2}\\&= (3.11) \text { in [8].} \end{aligned} \end{aligned}$$

Estimating the nonlinear part by the same analysis as in [8] yields

95 $$\begin{aligned} \begin{aligned} \frac{\textrm{d}}{\textrm{d}t} \Vert \tilde{u}(t)\Vert _{L^2}^2&\le \left( 1+ \Vert u_1(t)\Vert _{H^{\alpha }}^2 + \Vert u_2(t)\Vert _{H^{\alpha }}^2\right) \Vert \tilde{u}(t)\Vert _{L^2}^2, \\ \Vert \tilde{u}(0)\Vert _{L^2}^2&=0, \end{aligned} \end{aligned}$$

where the term in brackets is integrable, and hence implies pathwise uniqueness by Gronwall’s lemma.

Part (3) is then a consequence of the Yamada–Watanabe theorem (e.g. [22, Theorem 2.1]).

Next consider the following choice

96 $$\begin{aligned} \theta _k^N = \frac{1}{|k|^{\lambda }}\mathbbm {1}_{\{N \le |k| \le 2N\}}, \quad k \in \mathbb {Z}_0^d, N \in {\mathbb {N}}, \end{aligned}$$

for some $\lambda >0$. For any smooth divergence-free vector field $\phi$, Theorem 5.1 in [7] implies that

97 $$\begin{aligned} \lim _{N \rightarrow \infty } S_{\theta ^N}(\phi ) = \frac{3\nu }{5}\varDelta \phi \end{aligned}$$

in $L^2(\mathbb {T}^d)$. With this we shall prove the following result:

### Theorem 10

(cf. [8, Proposition 3.7] and [7, Theorem 1.4]) Let $\{u^N_0\}_N\subset L^2_\textrm{df}(\mathbb {T}^d)$ converge weakly in $L^2_\textrm{df}(\mathbb {T}^d)$ to some $u_0\in L^2_\textrm{df}(\mathbb {T}^d)$. Further let $u^N$ denote the unique strong solution to (77) associated to $\theta ^N$ defined in (96) starting at $u_0^N$. Then for every $\epsilon >0, p \ge 2$, $u^N$ converges in probability in the topology of $\mathcal {X}^{\epsilon , p}$ to the unique solution $u:= u(\cdot ; u_0, \nu )$ to

98 $$\begin{aligned} \begin{aligned} \partial _t u&= -\varLambda ^{2\alpha } u + \frac{3\nu }{5}\varDelta u + g_R(u)F(u),\\ u(0,\cdot )&= u_0. \end{aligned} \end{aligned}$$

### Proof

Recall from (86) that it holds

$$\begin{aligned} \sup _{t\in [0,T]} \Vert u^N(t)\Vert ^2_{L^2} + \int _0^T \left\| \varLambda ^{\alpha }u^N(t)\right\| ^2_{L^2} \textrm{d}t \le C\left( 1+\Vert u_0^N\Vert ^2_{L^2}\right) . \end{aligned}$$

Since $(u_0^N)_N$ is weakly convergent in $L^2(\mathbb {T}^d)$, it is bounded therein and we may thus find a constant *C* such that

99 $$\begin{aligned} \sup _{N \in {\mathbb {N}}} \left( \sup _{t\in [0,T]} \Vert u^N(t)\Vert ^2_{L^2} + \int _0^T \left\| \varLambda ^{\alpha }u^N(t)\right\| ^2_{L^2} \textrm{d}t\right) \le C \quad \mathbb {P}\text {-a.s.} \end{aligned}$$

Similar to previous estimates we may further find $q>1, \beta , \gamma >0$ such that

100 $$\begin{aligned} \sup _{N \in {\mathbb {N}}} {\mathbb {E}}\left[ \left( \Vert u^N\Vert _{L^{\infty }L^2} + \Vert u^N\Vert _{L^2H^{\alpha }} + \Vert u^N\Vert _{C^{\gamma }H^{-\beta }}\right) ^q\right] < \infty . \end{aligned}$$

Thus for every $\epsilon >0$ and $q \ge 2$ by [8, Lemma 3.3], the sequence of laws $(\mu ^N)_N, \mu ^N:= \textrm{Law}(u^N)$, is tight in $\mathcal {X}^{\epsilon , q}$ and, for *K* big enough, also in $\mathcal {X}_K$ (by (99)). By Prokhorov’s theorem, we may thus extract a subsequence $(\mu ^{N_n})_n$ which converges weakly to some probability measure $\mu$ on $\mathcal {X}_K$. For every $\phi \in C^{\infty }(\mathbb {T}^d)$, let the function $T^{\phi }:\mathcal {X}_K \rightarrow C([0,T];{\mathbb {R}})$ be defined by

$$\begin{aligned} \left( T^{\phi }f\right) (t)&:= \langle f(t), \phi \rangle _{L^2} - \langle u_0, \phi \rangle _{L^2} \\ {}&\quad - \int _0^t g_R(f(s)) \langle F(f(s)), \phi \rangle _{L^2} \text {d}s\\ {}&\quad - \int _0^t \langle f(s),- \varLambda ^{2\alpha } \phi + S_{\theta ^N}(\phi )\rangle _{L^2}\text {d}s, \end{aligned}$$

then due to Lemma 3.4 in [8] and (97), $T^{\phi }$ is continuous on $\mathcal {X}_K$. Following the same reasoning as in [8], it therefore holds $\mu =\delta _u$ where *u* is the unique solution to (98) which concludes the proof. $\square$

We conclude as in the proof of [8, Theorem 1.4]: Let $\epsilon >0,T\in (0,\infty )$ be fixed, then by hypothesis (H4) there exist $\nu>0, R >1$ such that

$$\begin{aligned} \sup _{u_0 \in \mathcal {K}\cap \mathcal {D}} \sup _{t \in [0,T]} \Vert u(t; u_0, \nu )\Vert _{L^2} \le R-1 \end{aligned}$$

hence $u(\cdot ;u_0, \nu )$ is a solution to the deterministic equation without cut-off. Let $u^R(\cdot ; u_0, \theta ^N, \nu )$ denote the solution to the cut-off equation (77) with $\theta ^N$ as in (96). By choice of $\mathcal {K}$ and due to Theorem 10 it holds

101 $$\begin{aligned} \begin{aligned}&\lim _{N \rightarrow \infty } \sup _{u_0 \in \mathcal {K}\cap \mathcal {D}} \mathbb {P}\left[ \sup _{t \in [0,T]} \left\| u^R(t;u_0,\theta ^N,\nu )\right\| _{H^{-\delta }}>R\right] \\&\le \lim _{N \rightarrow \infty } \sup _{u_0 \in \mathcal {K}\cap \mathcal {D}} \mathbb {P}\left[ \sup _{t \in [0,T]} \left\| u^R(t;u_0,\theta ^N,\nu )-u(t;u_0,\nu )\right\| _{H^{-\delta }}>1\right] =0 \end{aligned} \end{aligned}$$

and hence there exists *N* big enough such that uniformly in $u_0 \in \mathcal {K}\cap \mathcal {D}$ it holds

102 $$\begin{aligned} \mathbb {P}\left[ \sup _{t \in [0,T]} \left\| u^R(t;u_0,\theta ^N,\nu )\right\| _{H^{-\delta }}\le R\right] >1 - \epsilon , \end{aligned}$$

i.e. $u^R$ solves (77) without cut-off.
